# Carotid Intima-Media Thickness: Novel Loci, Sex-Specific Effects, and Genetic Correlations With Obesity and Glucometabolic Traits in UK Biobank

**DOI:** 10.1161/ATVBAHA.119.313226

**Published:** 2019-12-05

**Authors:** Rona J. Strawbridge, Joey Ward, Mark E.S. Bailey, Breda Cullen, Amy Ferguson, Nicholas Graham, Keira J.A. Johnston, Laura M. Lyall, Robert Pearsall, Jill Pell, Richard J. Shaw, Rachana Tank, Donald M. Lyall, Daniel J. Smith

**Affiliations:** 1 From the Institute of Health and Wellbeing (R.J.S., J.W., B.C., A.F., N.G., K.J.A.J., L.M.L., R.P., J.P., R.J.S., R.T., D.M.L., D.J.S.), University of Glasgow, United Kingdom; 2 School of Life Sciences, College of Medical, Veterinary and Life Sciences (M.E.S.B., K.J.A.J.), University of Glasgow, United Kingdom; 3 Health Data Research United Kingdom (R.J.S.); 4 Cardiovascular Medicine Unit, Department of Medicine Solna, Karolinska Institutet, Stockholm, Sweden (R.J.S.); 5 Division of Psychiatry, College of Medicine, University of Edinburgh, United Kingdom (K.J.A.J.).

**Keywords:** atherosclerosis, carotid artery, coronary artery disease, diabetes, type 2, genetics, association studies, intima-media thickness, obesity

## Abstract

**Objective:**

Atherosclerosis is the underlying cause of most cardiovascular disease, but mechanisms underlying atherosclerosis are incompletely understood. Ultrasound measurement of the carotid intima-media thickness (cIMT) can be used to measure vascular remodeling, which is indicative of atherosclerosis. Genome-wide association studies have identified many genetic loci associated with cIMT, but heterogeneity of measurements collected by many small cohorts have been a major limitation in these efforts. Here, we conducted genome-wide association analyses in UKB (UK Biobank; N=22 179), the largest single study with consistent cIMT measurements.

**Approach and Results:**

We used BOLT-LMM software to run linear regression of cIMT in UKB, adjusted for age, sex, and genotyping chip. In white British participants, we identified 5 novel loci associated with cIMT and replicated most previously reported loci. In the first sex-specific analyses of cIMT, we identified a locus on chromosome 5, associated with cIMT in women only and highlight *VCAN* as a good candidate gene at this locus. Genetic correlations with body mass index and glucometabolic traits were also observed. Two loci influenced risk of ischemic heart disease.

**ConclusionS:**

These findings replicate previously reported associations, highlight novel biology, and provide new directions for investigating the sex differences observed in cardiovascular disease presentation and progression.

## Abstract 2

Supplemental Digital Content is available in the text.

**Highlights**

- This is the largest genome-wide association study using consistent carotid intima-media thickness (cIMT) phenotyping to date.
- We identified 2 novel loci for average of mean cIMT values, 1 novel locus for average of maximum cIMT values, and 1 for average of mean cIMT values–average of maximum carotid intima-media thickness values.
- In the first sex-specific analysis of average of mean cIMT values we identified a women-specific locus.
- Genetic correlations with previous IMT meta-analyses, obesity, and glucometabolic traits were identified.
- *VCAN* and *SVIL* were highlighted as good candidate genes in 2 of the novel loci.

Atherosclerosis is the underlying cause of the majority of cardiovascular events and is characterized by vascular remodeling, incorporation of lipids into the vessel wall, and subsequent inflammation.^1,2^ Atherosclerosis is a systemic process that precedes clinical presentation of cardiovascular events, such as stroke, by decades. Indeed, evidence of vascular remodeling indicative of atherosclerosis has been observed as early as in adolescent age groups.^3^

**See accompanying editorial on page 297**

Atherosclerosis can be noninvasively assessed by ultrasound measurement of the carotid artery vessel wall, specifically the intima-media thickness (carotid intima-media thickness [cIMT]). In some cases, cIMT assessment is used for monitoring after cardiovascular events, such as stoke, but could also be useful for screening individuals at high risk of cardiovascular events. Currently, use is limited as it requires specialist equipment and training, and high-quality data analysis is laborious. Measurement of cIMT has been performed for research purposes, predominantly in cohorts recruited for the study of cardiovascular disease. Although undeniably useful, the use of clinical cohorts does not cover the whole spectrum of atherosclerotic burden in the population.

Genetic analyses of clinical cohorts have begun to identify single nucleotide polymorphisms (SNPs) associated with increased cIMT,^4–7^ which paves the way for better understanding of processes leading to cardiovascular events. A limitation for these studies (N=68 000) has been heterogeneity in recruitment and ultrasound methodology, which could lead to failure to detect some true genetic effects. In this respect, UKB (UK Biobank) provides an unprecedented opportunity to analyze IMT measurements in a very large cohort (N=22 000) with consistent recruitment and standardized cIMT measurements, analysis, and quality control.

We, therefore, set out primarily to identify genetic variants associated with cIMT in a large general population cohort. A secondary aim was to investigate the possibility of sex-specific genetic effects on IMT. Here, we demonstrate replication of previously reported associations, genetic correlations with cardiometabolic traits, novel biology and provide new directions for investigating the sex differences observed in cardiovascular disease presentation and progression.

## Methods

The individual-level data that underlie the findings of this study are available from UKB. Summary statistics resulting from this study are available from the corresponding author upon request.

### Study Population

The UKB study has been described in detail previously.^8,9^ In brief, UKB recruited ≈500 000 participants from the United Kingdom between 2006 and 2010. Participants attended 1 of the 22 recruitment centers across the United Kingdom where they provided a blood sample for DNA extraction and biomarker analysis and completed questionnaires covering a wide range of medical, social and lifestyle information. All participants provided informed consent, and the study was conducted in accordance with the Helsinki Declaration. Generic approval was granted by the National Health Service National Research Ethics Service (approval letter dated May 13, 2016, Ref 16/NW/0274) and the study conducted under UKB projects No. 7155 (Principal Investigator J. Pell) and No. 6553 (Principal Investigator D. Smith).

### Phenotyping

cIMT measurements were recorded at an imaging visit, 4 to 8 years after the recruitment. Starting in 2014, participants were invited to participate in an imaging assessment, which also included recording of anthropometric measurements and completion of questionnaires covering a wide range of medical, social, and lifestyle information (repeated from the baseline visit). cIMT phenotyping began in 2015, in a pilot phase, where n=2272 individuals were at imaged at 18 centers (with 8 centers accounting for 98% of the sample) with extensive manual quality control being conducted. Subsequently, manual quality control was deemed unnecessary, and all centers began recruiting and recording automated measurements (with 10 centers accounting for 93% of the sample). Details of the protocol are available at https://biobank.ctsu.ox.ac.uk/crystal/label.cgi?id=101. In brief, ultrasound measurements of the far wall, at 2 angles on each of the left and right, of the distal common carotid artery with automated software recording images and measurements of mean and maximum intima-media (UKB data fields 22670-22681). Recruitment for imaging is ongoing, but to date (2019), N=25 769 individuals have ultrasound measurements of the cIMT. Three measures were calculated: The average of 4 mean measures (2 for each of the left and right carotid arteries) was calculated for the mean (average of mean cIMT values [IMTmean]); the maximum IMT (average of maximum cIMT values [IMTmax]), where the largest of the 4 maximum IMT measures was used; the mean of 4 maximum measures (2 for each of the left and right carotid arteries) was calculated (IMTmean-max), for comparison with previous analyses.^4,6^ Where >1 value was missing due to poor quality of the image, the participant was excluded from analyses. Values were expressed in mm and natural log-transformed for normality before analysis.

In the follow-up assessment, anthropometric measures, lifestyle variables, medication, and disease history was again recorded including age, weight, waist circumference, hip circumference, waist:hip ratio, body mass index (BMI), systolic and diastolic blood pressure (SBP and DBP respectively), hypertension (defined as SBP ≥140 mm Hg or DBP ≥90 mm Hg or antihypertensive medication), probable type 2 diabetes mellitus (T2D, coding as per Eastwood et al^10^), ischemic heart disease (ISH, defined as heart attack or angina diagnosed by a doctor, data field 6150), stroke (defined as stroke diagnosed by a doctor, data field 6150). Corrected SBP and DBP, reflecting probable untreated levels, were calculated as per Ehret et al.^11^ These contemporary values were used in the analysis of cIMT.

### Genotyping

DNA was extracted from blood samples provided by participants, using standard protocols. Details of the UKB genotyping and imputation procedures have been described previously.^12,13^ Briefly, the full genetic data release (March 2018) was used for this study. Genotyping, preimputation quality control, imputation, and postimputation quality control were conducted centrally by UKB, according to standard procedures.

### Statistical Analyses

Descriptive statistics and Spearman rank correlations were conducted using Stata. Only individuals of self-reported white British ancestry were included in the genome-wide association study (GWAS) to maximize homogeneity. BOLT-LMM software was used to conduct genetic association analyses, to calculate heritability estimates and estimates of λ_GC_. IMTmean and IMTmax values were natural logarithm-transformed for normality, and genetic association analyses were conducted, adjusted for age, sex, and genotyping array (primary analysis) or age and genotyping array (secondary analyses). BOLT-LMM software models relatedness between individuals; therefore, genetic principle components were not included as covariates. SNPs were excluded if minor allele frequency <0.01, Hardy-Weinberg equilibrium *P*<1×10^-6^, or imputation score <0.3. Genome-wide significance was set at *P*<5×10^-8^, with suggestive evidence of association being set at *P*<1×10^-5^. After quality control, there were 22 179 participants with IMT and genetic data for analysis.

Genetic association results were visualized using FUMA software^14^ and LocusZoom software.^15^

A meta-analysis of sex-specific analyses was conducted to assess differences in effect sizes, using GWAMA software.^16,17^

For analysis of ISH and stroke, only unrelated individuals of self-reported white British ancestry who were not included in the cIMT GWAS were analyzed. Genetic association analysis was conducted in PLINK software,^18^ using a logistic model and assuming additive genetic effects and adjusting for age, sex, population structure (genetic principal components 1–8), chip, T2D, BMI, and current smoking.

### Linkage Disequilibrium and Genetic Correlations

Linkage disequilibrium (LD) between analyzed SNPs in each GWAS-significant locus was calculated and visualized in a random subset of 10 000 white British individuals (or 5000 individuals where the locus is computationally too large with 10 000 individuals) included in the cIMT subset, using Haploview (default settings).^19^

Genetic correlations between IMTmean and IMTmax and relevant cardiometabolic traits were calculated using previously published summary statistics and LD score regression.^20^ IMT summary statistics were provided by the CHARGE (Cohorts for Heart and Aging Research in Genomic Epidemiology) consortium (http://www.chargeconsortium.com/). Data on glycaemic traits were contributed by MAGIC (Meta-Analysis of Glucose and Insulin-Related Traits Consortium) investigators and were downloaded from www.magicinvestigators.org. T2D data were contributed by the DIAGRAM (Diabetes Genetics Replication and Meta-Analysis) consortium (http://diagram-consortium.org/downloads.html). Summary statistics for lipid traits were downloaded from the Global Lipids Genetics Consortium website (http://lipidgenetics.org/). Data on anthropometric traits were downloaded from the GIANT (Genetic Investigation of Anthropometric Traits) consortium website (http://portals.broadinstitute.org/collaboration/giant/index.php/Main_Page). Coronary artery disease data were downloaded from the CARDIoGRAMplusC4D (Coronary Artery Disease Genome Wide Replication and Meta-Analysis Plus the Coronary Artery Disease Genetics) consortium (http://www.cardiogramplusc4d.org/). Blood pressure data were provided by the International Consortium for Blood Pressure Genetics (https://www.ncbi.nlm.nih.gov/projects/gap/cgibin/study.cgi?study_id=phs000585.v1.p1).

### Data Mining

The GWAS catalog (https://www.ebi.ac.uk/gwas/, accessed 20190717) was used to identify previously reported associations with significant loci. All SNPs with at least suggestive evidence of association (*P*<1×10^-5^) within significantly associated loci were assessed for deleterious effect using the Ensemble variant effect predictor.^21^ GWAS-significant SNPs and those predicted by variant effect predictor to have at least moderate impact were assessed for effects on genotype expression patterns of nearby genes using the Genotype-Tissue Expression project.^22^ Functions of highlighted genes were explored using the National Centre for Biotechnology Information Gene platform (https://www.ncbi.nlm.nih.gov/gene/, accessed 20190717) and literature identified using National Centre for Biotechnology Information PubMed (https://www.ncbi.nlm.nih.gov/pubmed/, accessed 20190717).

## Results

Demographic characteristics of the UKB cIMT subset are presented in Table 1. The UKB cIMT subset consists of 48.3% men. Women were slightly younger (average 62.4 years) and were healthier (average BMI, 26.1 kg/m^2^ and SBP 132 mm Hg; % with hypertension, 53.8) and smoked less (% smokers, 3.1) compared with men (average, 63.9 years; BMI, 27.0 kg/m^2^; 141 mm Hg; % hypertension, 69.0; smokers, 4.2). Values for both IMTmean and IMTmax were lower in women than in men. Measurements of IMTmean and IMTmax were highly correlated (Spearman Rho=0.87, *P*<0.0001 [full cIMT subset], rho=0.868 *P*<0.0001 [men] and Rho=0.852 *P*<0.0001 [women]). IMTmean and IMTmax were significantly and positively associated with classical risk factors, including increasing age, obesity, and blood pressure (Table 2).

**Table 1.**
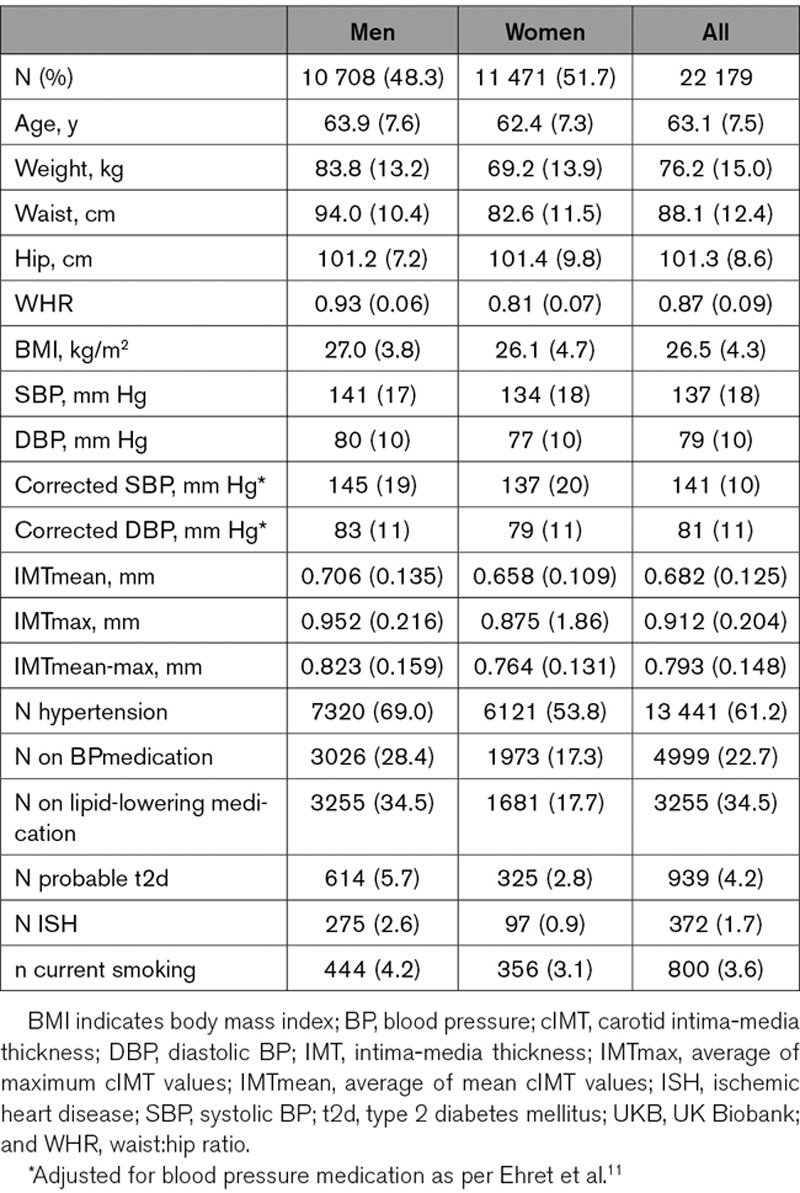
Demographics of the UKB Cohort With cIMT Measurements

**Table 2.**
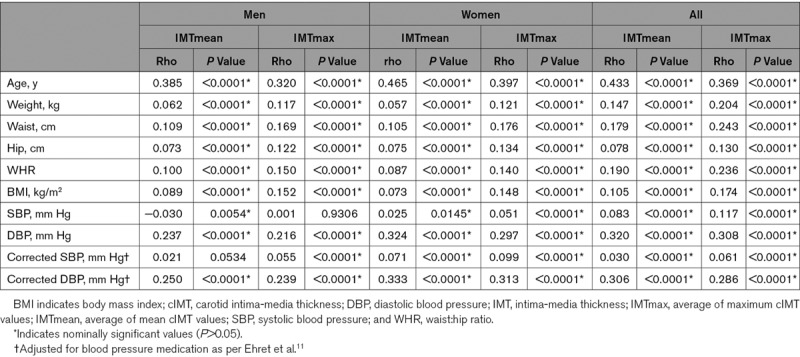
Correlations Between cIMT and Classical Cardiovascular Disease Risk Factors

### Primary Analysis: SNPs Associated With IMT_mean_ and IMT_max_

**Figure 1.**
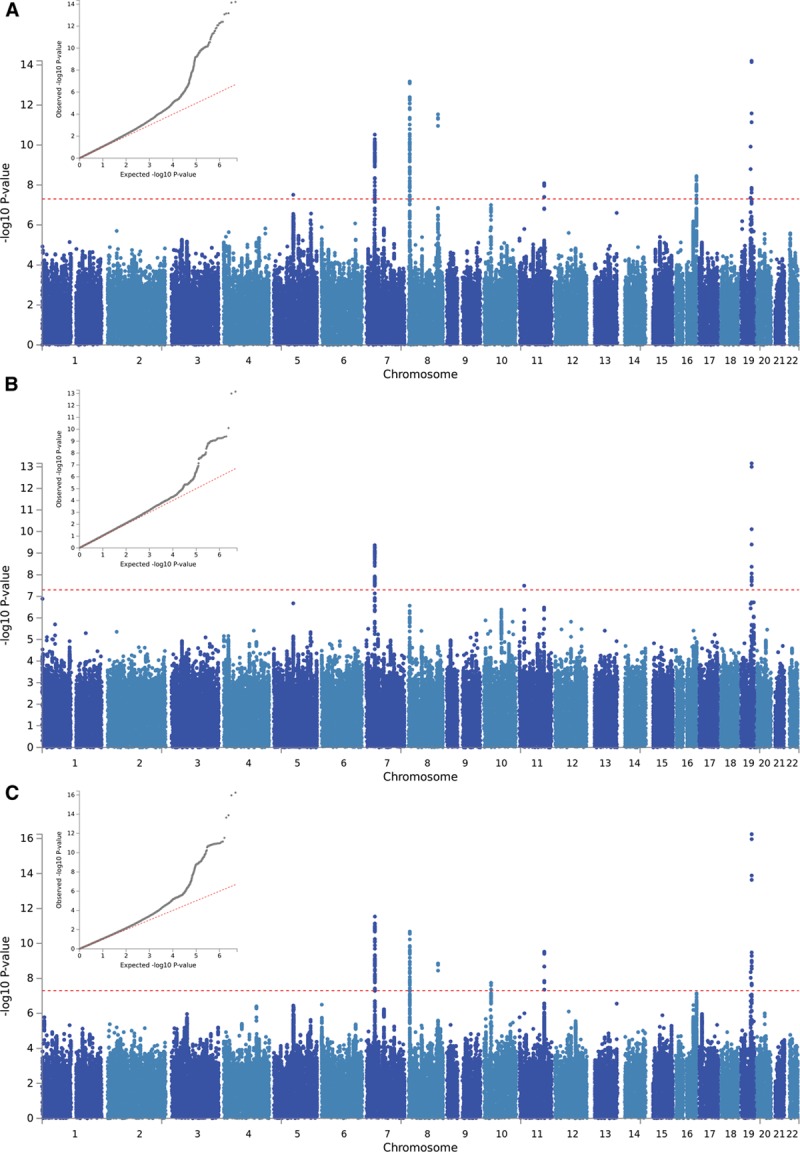
**QQ and Manhattan plots of results of the genome-wide association study of (A) average of mean carotid intima-media thickness (cIMT) values (IMTmean), (B) average of maximum cIMT values (IMTmax), and (C) IMTmean-max.**

Manhattan and QQ plots of the GWAS results are presented in Figure 1. There was some evidence of inflation for both IMTmean and IMTmax (λ_GC_=1.15 and 1.10, respectively); however, this is likely due to polygenicity rather than population structure (linkage disequilibrium score regression intercept [SE]=1.03 [0.03] for both phenotypes). Figure I in the online-only Data Supplement demonstrates the genetic homogeneity of the participants included in the analyses.

GWAS-significant evidence of association with IMTmean was observed for 176 SNPs in 8 loci (Figure 1A), and 76 SNPs in 3 loci demonstrated GWAS-significant evidence of association with IMTmax (Figure 1B). The lead SNP for each locus is provided in Table 3. As BOLT-LMM includes neighboring SNPs in the model (essentially conditioning on other SNPs in the region), each locus reported here is independent and contains only a single signal. Effect sizes of all lead SNPs were comparable with those previously reported (β, 0.0091–0.441).^6^

**Table 3.**
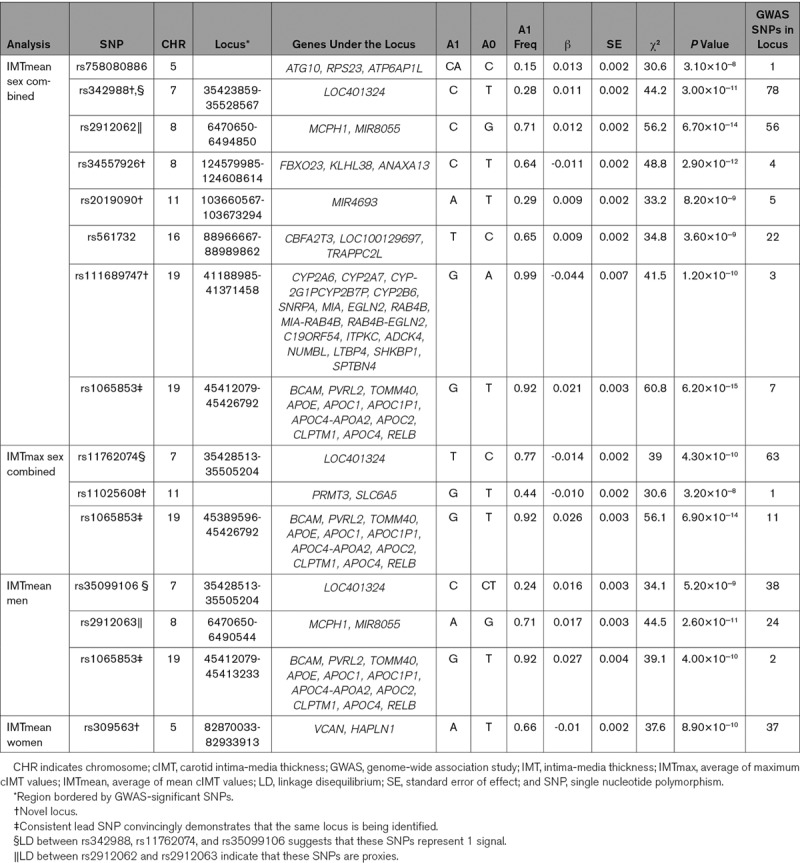
Lead SNPs in Loci Associated With cIMT

**Figure 2.**
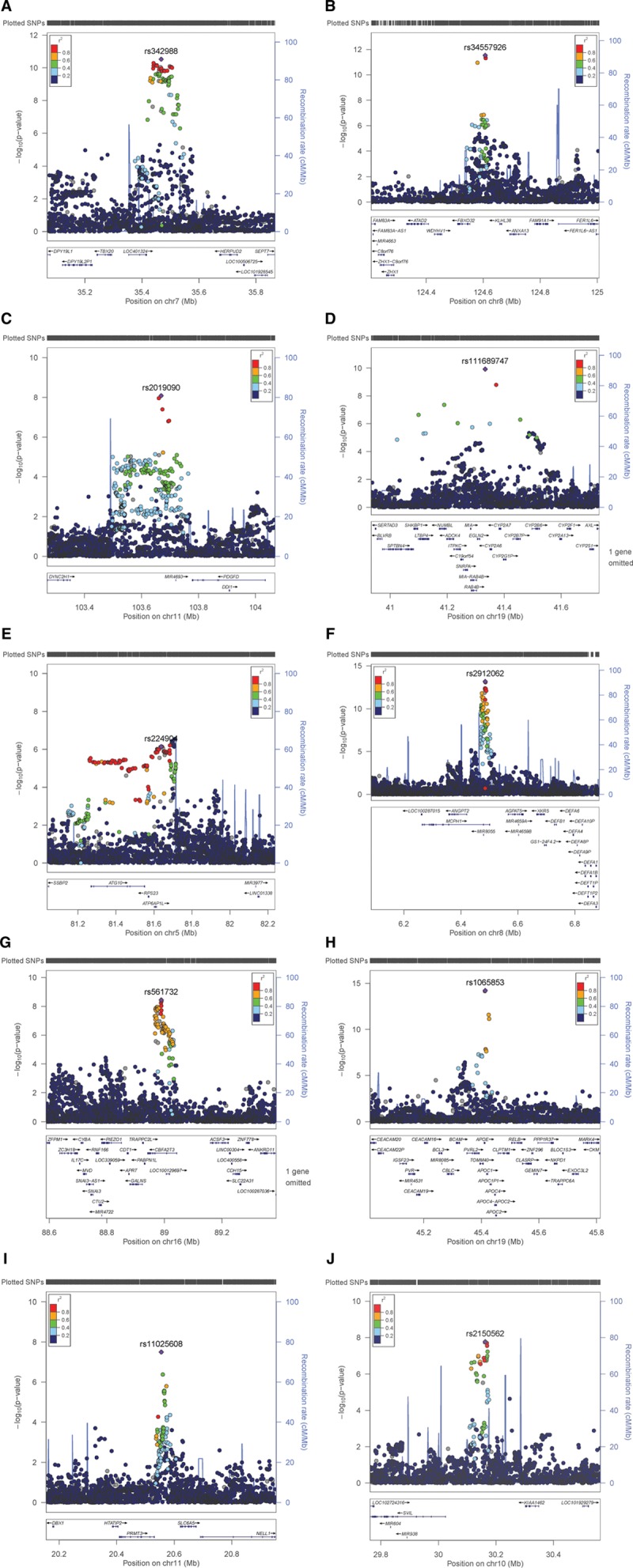
**Regional plots for novel average of mean carotid intima-media thickness (cIMT) values (IMTmean)–associated loci on (A) chromosome (Chr) 7 (rs342988), (B) Chr8 (rs34557926), (C) Chr11 (rs2019090), (D) Chr19 (rs111689747) and known IMTmean-associated loci on (E) Chr5 (rs224904, instead of lead SNP rs758080886), (F), Chr8 (rs2912062), (G) Chr16 (rs561732), (H) Chr19 (rs1065853) as well as (I) the novel locus for average of maximum cIMT values on Chr11 (rs11025608).** The lead single nucleotide polymorphism (SNP) is indicated by a purple diamond. Linkage disequilibrium (LD; *r*^2^) between other SNPs and the lead SNP is indicated by color. Gray indicates LD is not known.

Of the 8 loci significantly associated with IMTmean identified here, 4 are novel (chromosome 7, lead SNP rs342988; chromosome 8 [124.6 Mb], rs34557926; chromosome 11, rs2019090; chromosome 19 [41.3 Mb], rs111689747; Figure 2A through 2D), and 4 have previously been reported (Figure 2E through 2H).

In the previously reported loci, on chromosome 5 there are a handful of SNPs which reach GWAS-significant evidence for association with IMTmean, where the lead SNP (rs758080886) is located 3.417 kb from the previously reported lead SNP rs224904.^6^ rs758080886 is not available in the reference panel used by LocusZoom^15^ and is, therefore, not plotted; hence, this region is represented by the previously reported SNP, rs224904. The LD between rs75808088 and rs224904 is moderate (*r*^2^=0.44); therefore, rs224904 is not a good proxy; however, this is the same for all SNPs in the locus (Figure II in the online-only Data Supplement). On chromosome 8, rs2912062 is 0.738 kb from the previously reported lead, rs2912063, and in high LD (*r*^2^=0.98), demonstrating that these SNPs represent the same signal. On chromosome 16, the lead SNP, rs561732, is 22.3 kb from the previously reported lead, rs844396, with moderate LD (*r*^2^=0.66). The chromosome 19 lead here, rs1065853, is 1.15 kb from the reported rs7412 but with almost complete LD (*r*^2^=0.99). The novel chromosome 19 locus is ≈4 Mb from this signal, and although long-range LD is possible, the calculated LD between rs111689747 and rs1065853 or rs7412 does not support this possibility (*r*^2^=0).

There were 3 GWAS-significant loci for IMTmax. Of these, 2 overlap with those for IMTmean: the lead SNPs for IMTmean and IMTmax on chromosome 7 (rs342988 and rs11762074, respectively) are 26.9 kb apart with LD of *r*^2^=0.77. For chromosome 19, the locus not only overlaps, but the lead SNP is the same for both traits. The chromosome 11 for IMTmax (20.5 Mb) is distinct from that for IMTmean (103.6 Mb; Figure 2I).

When considering SNPs significantly associated with IMTmean, the direction of effects on IMTmax are consistent with those for IMTmean, and the magnitude is similar (Table I in the online-only Data Supplement). The same is true for the chromosome 11 IMTmax SNP, rs11025608, which shows a similar effect size and direction in IMTmean (β, −0.008; [SE] 0.002; *P*=1.6×10^-6^). Therefore, further analyses focused on IMTmean for more robust comparisons with previous studies.

### UKB IMT GWAS and the CHARGE Consortium IMT GWAS Meta-Analysis

The largest GWAS meta-analysis of cIMT previously published by the CHARGE consortium^6^ included 68 962 individuals from 30 studies with a variety of recruitment strategies, inclusion criteria, and measurements of cIMT. In this study, IMTmean-max was analyzed. The range of average ages was 37.7 to 78.8 years, and average IMTmean-max was 0.50 to 1.13 mm. The single largest study within the meta-analysis included 8663 individuals (approximately evenly split between men and women).

The UKB study (N=22 179) is within the age and cIMT ranges reported by the CHARGE consortium, albeit on the younger and healthier end of the spectrum. IMTmean-max in UKB was highly correlated with IMTmean (Rho=0.964; *P*<0.0001) and IMTmax (Rho=0.931; *P*<0.0001). GWAS results of IMTmean-max (Figure 1C) demonstrated similar results to those for IMTmean, with 6 loci being identified. Of these, 5 had the same lead SNP as for IMTmean, and 1 locus on chromosome 10 was novel (lead SNP rs2150562; Table 3; Figure 2J). In the analysis of IMTmean, this locus reached suggestive significance (β=0.008, [SE]=0.002, *P*=1.5×10^-6^). All loci reaching GWAS-significance (*P*<5×10^-8^) for IMTmean or IMTmax demonstrated at least suggestive (*P*<1×10^-6^) significance with IMTmean-max (Table I in the online-only Data Supplement).

The CHARGE meta-analysis reported 11 robustly associated loci,^6^ of which 9 lead SNPs were available in UKB (Table II in the online-only Data Supplement). Seven of these demonstrated significant (*P*<0.05) associations with IMTmean and IMTmean-max, with consistent effect directions, when compared with the previous report^6^ (Table II in the online-only Data Supplement). One SNP demonstrated a nonsignificant association, and one demonstrated a significant association but inconsistent direction (Table II in the online-only Data Supplement), for both IMTmean and IMTmean-max.

Of the lead SNPs identified in UKB, 11 were available in the CHARGE meta-analysis (Table III in the online-only Data Supplement). Of these, 10 were significant (*P*<0.05) with consistent effect directions to those reported in UKB. One lead SNP was not significant. Effects sizes were generally 2- to 3-fold larger in UKB than in the CHARGE meta-analysis. The effect sizes of the cIMT-associated lead SNPs are summarized in Figure III in the online-only Data Supplement.

### Secondary Analyses: Sex-Specific Genetic Effects on IMT

**Figure 3.**
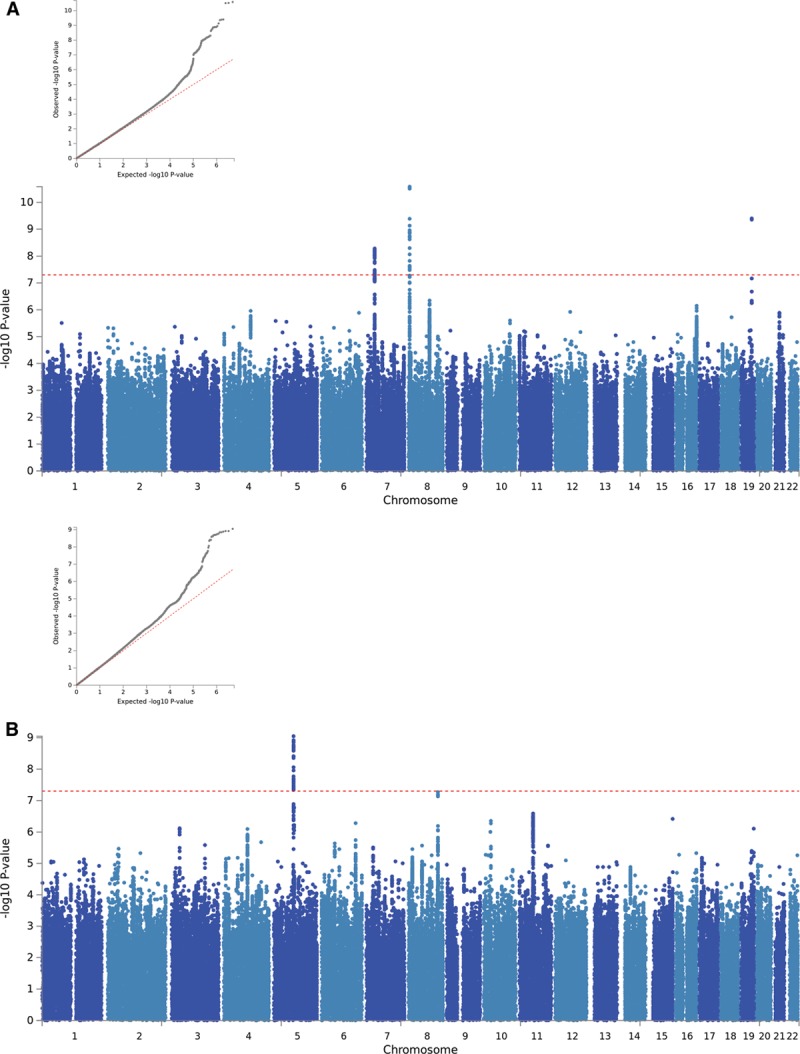
**QQ and Manhattan plots of results of the genome-wide association study of intima-media thickness mean in (A) men only and (B) women only.**

**Figure 4.**
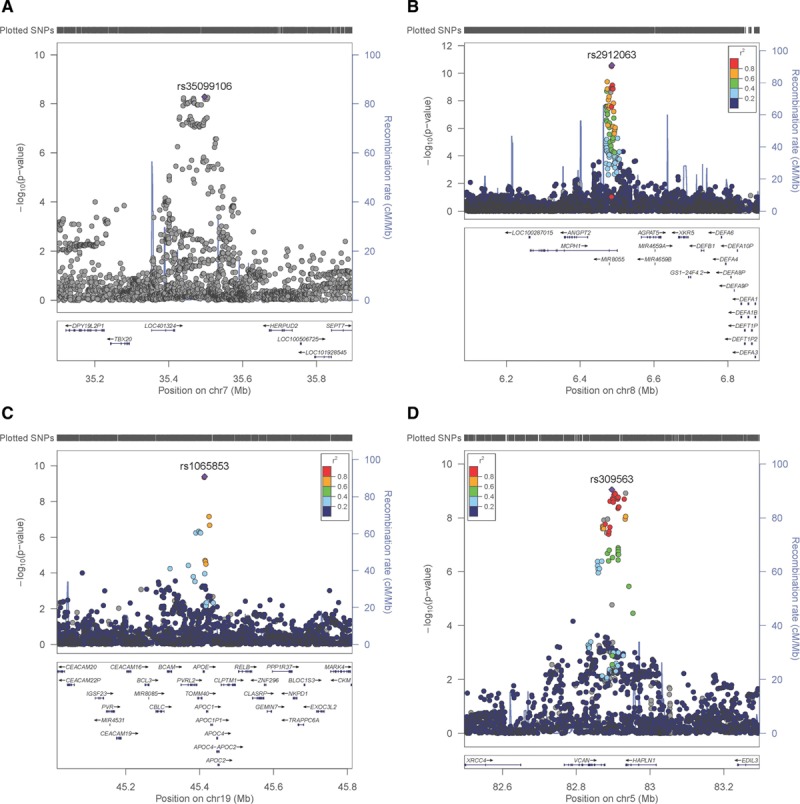
**Regional plots for sex-specific average of mean carotid intima-media thickness value–associated loci.** Men-only loci on (**A**) chromosome (Chr) 7 (rs35099106), (**B**) Chr8 (rs2912063), (**C**) Chr19 (rs1065853) and the women-only locus on (**D**) Chr5 (rs309563). The lead single nucleotide polymorphism (SNP) is indicated by a purple diamond. Linkage disequilibrium (LD; *r*^2^) between other SNPs and the lead SNP is indicated by color. Gray indicates LD is not known.

Sex-specific analyses suggest that the genetic variants associated with IMTmean in men and women are distinctly different (Figure 3): In men-only analyses (Figure 3A), 3 GWAS-significant loci were identified on chromosome 7 (rs35099106), chromosome 8 (6.4 Mb, rs2912063), and chromosome 19 (rs1065853), all of which overlap with those identified in the primary (sex combined) analysis (Table 3 and Figure 4). The chromosome 7 lead SNP is in strong LD with that for IMTmax (rs11762074; *r*^2^=0.97) but only moderate LD with the sex-combined IMTmean lead SNP (rs342988; *r*^2^=0.64). The lead SNPs on chromosome 8 and 19 are consistent either with the primary analysis or with previously reported lead SNPs.

In an analysis of women (Figure 3B), only a locus on chromosome 5 was GWAS-significant (Table 3 and Figure 4). The lead SNP for this locus, rs309563, is ≈1.2 Mb from, and demonstrated no LD with, the lead sex-combined SNP for IMTmean (rs758080886, *r*^2^=0) or the previously reported lead SNP for this locus (rs224904, *r*^2^=0, Figure II in the online-only Data Supplement), suggesting that it is a separate locus.

Effect directions for SNPs with suggestive evidence of association were consistent between men and women. All 3 loci identified in the men-only analysis have concordant effect directions (same direction of effect, but different sizes between the sexes^23^), where effect sizes were halved in women but at least nominal significance being observed for most SNPs and suggestive significance noted for the chromosome 19/*APOE* locus (Table IV in the online-only Data Supplement). In contrast, the chromosome 5 locus identified in the women-only analysis is a single-sex effect locus,^23^ that is to say it demonstrates nonsignificant associations in men (Table IV in the online-only Data Supplement). In all 4 loci, SNPs demonstrated significant heterogeneity between men and women (with I^2^>0.75 and *P*<0.047; Table IV in the online-only Data Supplement).

### Impact of cIMT-Associated Variants on ISH and Stroke in UKB

Demographic characteristics of the individuals included in the analyses of ISH and Stroke are presented in Tables IX and X in the online-only Data Supplement, respectively. Unsurprisingly, ISH and stroke cases had higher age, BMI, and rates of hypertension and blood pressure and lipid-lowering medication. Two IMT-associated SNPs (rs758080886 and rs1065853) were nominally (*P*<0.05) associated with stroke (Table XI in the online-only Data Supplement) but did not withstand multiple testing correction (13 SNPs, *P*<0.0038). Three IMT-associated SNPs were associated with ISH, 2 of which survived multiple testing correction (Table XI in the online-only Data Supplement). The effects of rs2019090 and rs1065853 were in the expected direction, with the allele associated with increased IMTmean measures being associated with increased risk of ISH.

### Genetic Correlations With Cardiovascular Phenotypes and Risk Factors

UKB IMTmean, IMTmax, and IMTmean-max GWAS demonstrated significant genetic correlations with the CHARGE GWAS IMTmean meta-analyses (regression coefficient [rg]=0.74–0.82, Table 4).^4,6^ As the individuals included in the IMTmean, IMTmax, and IMTmean-max analyses overlap completely was not possible to compare these analyses. Significant positive genetic correlations were observed between IMTmean and total obesity (BMI), T2D, fasting glucose, and insulin (Table 4). For IMTmax, positive correlations with total and central obesity (BMI and waist:hip ratio adjusted for BMI respectively), T2D, fasting glucose, and rheumatoid arthritis were observed (Table 4). It was surprising to note that a significant positive association with HDL (high-density lipoprotein) and a significant negative association with fasting insulin was observed. It is worth noting that the fasting insulin adjusted for BMI demonstrated the expected direction or correlation, suggesting that obesity influences the relationship between IMTmax and fasting insulin levels. The significance of the positive correlation between IMTmax and HDL is unclear. Genetic correlations for IMTmean-max were similar to those for IMTmean and IMTmax (Table 4).

**Table 4.**
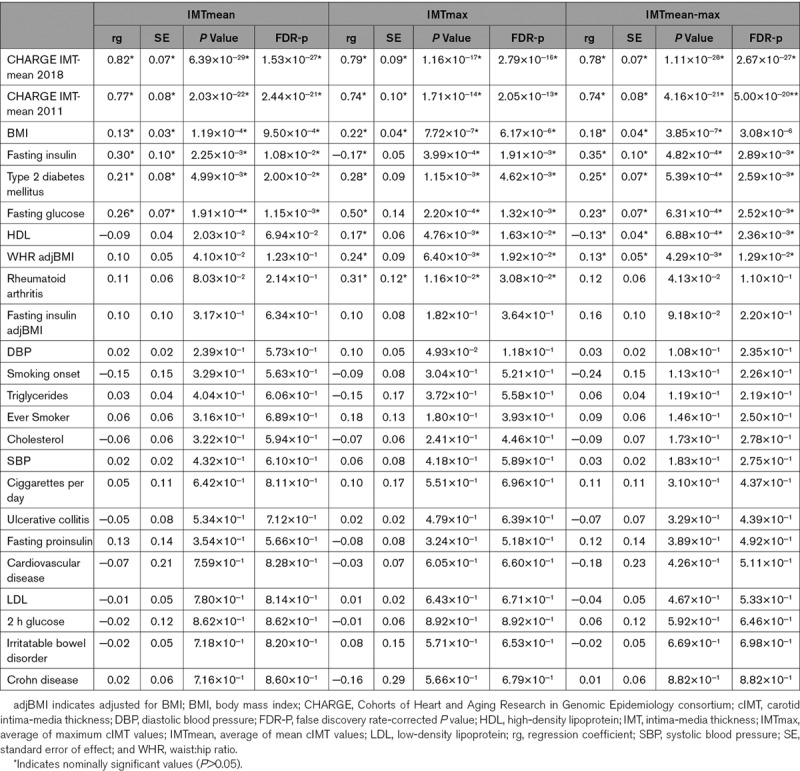
Genetic Correlations Between IMTmean, IMTmax, and Cardiometabolic Traits and Risk Factors

In men, similar to the sex-combined results, IMTmean was positively genetically correlated with the CHARGE IMTmean meta-analyses, total obesity, fasting glucose, and fasting insulin. In women, only the genetic correlations with the CHARGE meta-analyses survive false discovery rate correction (Table V in the online-only Data Supplement).

### Data Mining

The GWAS catalog was explored (using lead SNPs and locus positions, as per Table 3) to identify previous associations with the cIMT-associated loci (Table VI in the online-only Data Supplement). Chromosome 7 and chromosome 8 (6.4 Mb) have previously been associated with DBP,^24,25^ whereas chromosome 11, chromosome 13, and both chromosome 19 loci have previously been associated with coronary artery disease^26–28^ or pulse pressure^29^ or cIMT.^6^ The novel chromosome 10 locus has previously been associated with height.^30^ Atherosclerosis is considered a condition requiring both fatty deposits and inflammation in the vascular wall. Therefore it was interesting to note the reported associations of chromosome 8 (6.4 Mb) and chromosome 11 with immune response^31^ or immune components^32^ as well as the chromosome 19 (45 Mb) locus with lipid (and other biomarker) levels^33–50^ and chromosome 7 with fatty liver disease.^51^

The chromosome 19 locus includes the apolipoprotein gene cluster (including *APOE*), which has been the focus of much research into lipid levels, cardiovascular disease, and Alzheimer disease. In contrast, previous associations at the chromosome 5, chromosome 8 (124 Mb) and chromosome 16 loci have no obvious relevance to cIMT. For the vast majority of previous associations with relevant traits (Table VI in the online-only Data Supplement), the effects on cIMT were in the expected direction: the alleles associated with increased DBP, SBP, pulse pressure, total cholesterol, low-density lipoprotein, APOE and lipoprotein A levels, and lipoprotein phospholipase A2 activity were associated with increased cIMT, as were the alleles associated with decreased HDL levels. The only unexpected finding was that the rs7412 allele associated with decreasing triglyceride levels^47^ was associated with increased cIMT; however, the authors suggest that the skewed distribution of lipids in those homozygous for this allele might cause inflation of test statistics^47^ and that this should be considered when interpreting results.

Of SNPs within significantly associated loci showing at least suggestive evidence of association with IMTmean, 13 SNPs were predicted^21^ to have functional or coding effects (Table VII in the online-only Data Supplement). Lead SNPs and those predicted by variant effect predictor to have functional or coding effects were assessed for evidence of genotype-specific effects on gene expression levels (expression quantitative trait loci) in the Genotype-Tissue Expression project dataset (Table VIII in the online-only Data Supplement). Only rs2019090 on chromosome 11 demonstrated expression quantitative trait loci in a tissue of obvious relevance, namely the aorta, where the cIMT-increasing allele was associated with increased *RP11-563P16.1* levels. Lack of knowledge about this gene's role precludes interpretation of this finding.

### Candidate Genes

Of the genes highlighted by expression quantitative trait loci analysis (though in a tissue of unclear relevance), *APOE* (chromosome 19) has been widely studied in cardiovascular (and other, notably Alzheimer) diseases,^52^ whereas the function of *MCPH1-AS1(CTD-2541M15.3*;chromosome 8) is thought to be as a regulator of *MCPH1*, a DNA damage response gene with no obvious role in vessel wall biology. Similarly, *CBFA2T3* (chromosome 16) has documented roles in cancer biology as a transcriptional repressor, but again, there is no obvious role in vascular biology.

*VCAN* (chromosome 5, women-only locus) is potentially an interesting candidate gene. Its encoded product, versican, is a chondroitin sulfate proteoglycan present in the adventitia and intima of normal blood vessels (reviewed in Wight and Merrilees^53^). Versican protein levels have been shown to increase dramatically during progression of vascular diseases including atherosclerosis.^53^ Versican exists as a variety of protein isoforms of different sizes, due to alternative splicing, a variety of post-translational modifications, and proteolytic cleavage.^53^ The diverse effects of versican in vascular pathology are likely determined by the balance of different sized isoforms and partners in complexes^53^: larger molecules are better able to bind and thus retain low-density lipoprotein L in the vessel wall; loss of the largest versican isoform has been observed in aortic aneurysms; various cytokines promote different sized versican entities or versican degradation; smaller versican isoforms could act directly as mitogens for arterial smooth muscle cells; and the smallest isoform of versican influences elastic fiber formation and, therefore, vessel function. Therefore, it is interesting to note that the lead SNP for the women-only locus is an expression quantitative trait loci for a versican antisense molecule, *VSCAN1-AS1*, in testes, suggesting yet another mechanism regulating versican expression, although the role of versican in the testes is unknown. Full understanding of versican biology is required before the impact of this SNP is predictable.

*SVIL* in the novel chromosome 10 locus encodes supervilin, which is a cytoskeleton-regulating protein that influences platelet reactivity.^54^ Supervillin levels have been associated with platelet function in response to shear stress (in humans and mice), with increased levels of supervillin being associated with reduced thrombus formation^54^; therefore, it was interesting to note that the allele associated with increased IMTmean-max measures (rs2150562-A) was associated with reduced *SVIL* levels in the aorta (Table VIII in the online-only Data Supplement) but not with heart disease or stroke outcomes (Table IX in the online-only Data Supplement). However, the lead SNP for IMTmean-max, rs2150562 is ≈350 kb upstream of a variant, which has been associated with platelet thrombus formation, rs7070678^54^ and LD between rs2150562 and rs7070678 was 0 (*R*^2^). This suggests that the signal for IMTmean-max is distinct from that associated with platelet function. There are multiple isoforms of supervillin, with incompletely characterized functions.^55^ However, there is evidence that at least 1 isoform influences cell migration and proliferation,^55^ which are of relevance to atherosclerosis pathology. How the genetic variants influence Supervillin isoforms is as yet unclear.

## Discussion

Here, we present results from a GWAS of the largest single study of cIMT to date, and the first sex-specific analysis of cIMT. We identified 11 loci (5 of which were novel) associated with cIMT (mean, max or mean-max) and 1 locus specific for IMTmean in women. We also found genetic correlations with obesity, glucometabolic, and lipid traits, which suggest differences between sexes.

Many studies have reported the utility of cIMT measurements as predictors of cardiovascular disease (reviewed by Katakami et al^56^), although whether predictive power is independent of traditional risk factors is still unclear. A large part of the discrepancies in the literature is likely due to the different protocols used (which part of the carotid artery is measured, whether plaque is included or not, whether mean or plaque [defined by a specific maximum value] is used) in the analyses. It is worth noting that IMTmean-max is most often analyzed, with IMTmax rarely being analyzed a quantitative trait, rather this measure is normally used to define the presence of plaque (as described^6^). Although plaque is clearly a relevant phenotype, we used the better-powered continuous IMTmax values to explore the possibility of different genetic regulation and potentially different mechanisms influencing mean and maximum cIMT measures.

Indeed it has been reported that IMTmean and IMTmax differ in their predictive value.^56^ Therefore, the partial overlap in loci associated with IMTmean and IMTmax is of interest. For the most part, SNPs influencing one phenotype also influence the other, but the relative importance of each locus (or mechanism) differs. This hints at differences in biology between the 2 measures, which is perhaps not surprising: overall increases in vascular wall thickness could indicate general vascular dysfunction, which is a component of high blood pressure for example. Local increases in vascular wall thickness are likely indicative of plaque formation, which precede vessel occlusion and ischemic events.

Our results demonstrate that only a minority of SNPs associated with cIMT measures have significant effects on cardiovascular end points, namely ISH or stroke, which is consistent with previous reports.^6^ It was reassuring to note that the 2 SNPs with effects on ISH had the expected effects, with increased cIMT being associated with increased risk of ISH and that one of these SNPs was previously associated with myocardial infarction.^27^

Sex-specific differences in cardiovascular disease are well established; therefore, the distinctly different IMTmean GWAS results for men compared with women were intriguing. The existence of a women-only locus, whereas the men-only analysis largely resembled the sex-combined analysis, is unexpected. It suggests that the sex-combined results could be driven by the men. This is unlikely given the fairly balanced sex distribution of the sample (48% men versus 52% women); however, the larger variability in measures in men compared with women could cause this effect. It has been observed that sex differences in IMT measures can be attributed to sex differences in risk factors, such as BMI and blood pressure.^57^ Despite UKB being ≈20 years older than the subjects in that study, similar sex differences in risk factors are observed here. It is also worth noting that menopause status is associated both with cIMT measures and cardiovascular risk factors.^58,59^ Menopause status was not considered in the study, as direction of effect is unclear. However, this factor would be worth considering for future studies of sex differences.

Another noteworthy finding is the stronger effect of *APOE* rs7412 in men compared with women (β=0.027 versus 0.016, respectively). To our knowledge, this is the first demonstration of a sex effect of *APOE* variation in a cardiovascular disease phenotype, which is of value when considering the relatively nascent concept of an aging, sex, and *APOE* triad.^60^ Sex-specific differences in *APOE-e* genotypes associations with Alzheimer disease have been noted (summarized by Fisher et al^61^), with female carriers of *APOE-e3/e4* demonstrating increased risk of Alzheimer disease and faster decline than their male counterparts. Indeed, this finding (a variant that increases risk of atherosclerosis for men and Alzheimer disease for women) supports the hypothesis that women have higher rates for Alzheimer disease than men, at least partly because men with cardiovascular disease die earlier than women; therefore, in the at-risk age-range for Alzheimer disease, men have lower cardiovascular risk than women.^61^

In line with known epidemiology and previous genetic findings,^62^ the genetic correlations between IMTmean, IMTmax, or IMTmean-max and obesity and glucometabolic traits are unsurprising. It is worth noting that genetic correlations between coronary artery disease (ie, cardiovascular end points) and fasting insulin or glucose are quite strong but not reported due to their unimpressive *P* values (rg 0.23 [se 0.11], *P*=0.041 and 0.14 [0.09] *P*=0.095 respectively).^63^ Rheumatoid arthritis demonstrates weak and nonsignificant associations with coronary artery disease (−0.06 [0.08], *P*=0.438), but a moderate and nominally significant association with IMTmax. Taken together these findings suggest different mechanisms are important during subclinical atherosclerosis compared with clinically evident cardiovascular disease. The lack of genetic correlations between IMTmean and lipid traits is unexpected but may reflect the relative health, adherence to statin therapy, or healthy diet of the general population sample of UKB. Similarly, we cannot exclude the possibility that the relative health or use of statin therapy contributes to the unexpected positive correlation between IMTmax and HDL. As this is the first time IMTmax has been analyzed using a GWAS and genetic correlations, it is not possible to validate or further explore this finding.

In comparison with the CHARGE consortium cIMT meta-analysis, this study was smaller (N=22 000 versus N=68 000) but used a phenotype that was recorded in a consistent manner in all individuals, reducing heterogeneity in measurements. This is likely the reason for identifying loci not reported by CHARGE as well as the larger effect sizes reported here and is in line with previous observations (eg, the C4D^26^ and CARDIoGRAM findings^64^). In light of the recently identified issues with some genotyping in UKB (exemplified by Wei et al^65^), quality control measures for lead SNPs are reported in Table XII in the online-only Data Supplement. For all but one lead SNP, these measures indicate no issues. Only rs11025608 should be viewed with caution until replication of this signal has been conducted.

It should be noted that the location of measurements relative to the bifurcation is not specified and that there is some evidence of inflation of the GWAS statistics, for which there are a number of possible explanations. Polygenicity is an obvious explanation and highly plausible, as it is observed most complex traits. Although the pilot phase cIMT data (n=2500) underwent manual quality control procedures, the full IMT dataset (N=22 000) did not. Comparisons between the quality controlled and non-quality controlled data in the pilot study show very high correlation (IMTmean rho=1.0, IMTmax rho=0.98); however, the possibility of noise in measurements cannot be excluded. Despite this limitation, the consistent effect sizes and directions of most previous IMT loci suggest that this study is valid.

In conclusion, we have conducted a GWAS of cIMT (mean and maximum measures) in the largest single study sample to date, in which we identified 4 novel loci and validated 7 of 11 previously reported loci. We also report the first sex-specific analysis of IMT, in which we identified a novel women-only locus. Genetic correlations with obesity and glucometabolic traits were observed, and *VCAN* was highlighted as a plausible candidate gene in the women-only locus. Overall, our findings represent an important stimulus for the further elucidation of mechanisms of vascular pathology, particularly differences between men and women, and could contribute to stratified medicine approaches.

## Acknowledgments

We thank all participants and staff of the UKB study (UK Biobank). This work uses data provided by patients and collected by the National Health Service as part of their care and support. The assistance of Rosie Brown (University of Glasgow) in producing forest plots is highly appreciated

## Sources of Funding

The UKB (UK Biobank) was established by the Wellcome Trust, Medical Research Council, Department of Health, Scottish Government, and Northwest Regional Development Agency. UKB has also had funding from the Welsh Assembly Government and the British Heart Foundation. Data collection was funded by UKB. R.J. Strawbridge is supported by a UK Research and Innovation-Health Data Research UK Fellowship (MR/S003061/1). J. Ward is supported by the John, Margaret, Alfred and Stewart Sim Fellowship for depression research from the Royal College of Physicians of Edinburgh (173558). A. Ferguson is supported by a Medical Research Council (MRC) Doctoral Training Programme Studentship at the University of Glasgow (MR/K501335/1). K.J.A. Johnston is supported by an MRC Doctoral Training Programme Studentship at the Universities of Glasgow and Edinburgh. D.J. Smith acknowledges the support of a Lister Prize Fellowship (173096) and MRC Mental Health Data Pathfinder Award (MC_PC_17217).

## Disclosures

None.

## Supplementary Material
