# Supplementary material for: Carotid Intima-Media Thickness: Novel Loci, Sex-Specific Effects, and Genetic Correlations With Obesity and Glucometabolic Traits in UK Biobank
Source: Arterioscler Thromb Vasc Biol. 2019 Dec 5;40(2):446–61. doi: 10.1161/ATVBAHA.119.313226 (PMC6975521; doi:10.1161/ATVBAHA.119.313226)
Supplement: Supplementary file 1 [file atv-40-446-s001.pdf]

SUPPLEMENTAL MATERIALS

Carotid intima-media thickness: novel loci, sex-specific effects and genetic correlations with obesity and glucometabolic traits in UK Biobank

Rona J. Strawbridge PhD[1,2,3], Joey Ward MSc[1], Mark E.S. Bailey PhD[3], Breda Cullen PhD[1], Amy Ferguson PhD[1], Nicholas Graham MBChB[1], Keira J.A. Johnston MSc[1,3,4], Laura M. Lyall PhD[1], Robert Pearsall DPhil[1], Jill Pell MD[1], Richard J Shaw PhD[1], Rachana Tank MSc[1], Donald M. Lyall PhD[1] and Daniel J. Smith MD[1]

[1]Institute of Health and Wellbeing, University of Glasgow, Glasgow, UK.

[2]Health Data Research UK

[3]Cardiovascular Medicine Unit, Department of Medicine Solna, Karolinska Institutet, Stockholm, Sweden

[3]School of Life Sciences, College of Medical, Veterinary and Life Sciences, University of Glasgow, Glasgow, UK

[4]Division of Psychiatry, College of Medicine, University of Edinburgh, Edinburgh, UK

***** Corresponding author:

Dr Rona J. Strawbridge (rona.strawbridge@glasgow.ac.uk, +44 (0)141 330 3842)

1 Lilybank Gardens, Glasgow, G12 8RZ, UK

Supplemental Figure I: Plot of first two principle genetic components (PGCs), demonstrating the homogeneity of the white British participants in the UK Biobank IMT sample.

Supplemental Figure II: LD between SNPs with suggestive evidence of association in two loci on Chr5. Highlighted SNPs are A) Lead SNP for GW-significant locus for IMTmean on Chr5 (rs758080886), B) previously reported lead SNP (rs22490) and C)

lead SNP for the women-only locus for IMTmean (rs309563). Inset, zoomed in LD plot of only these three SNPs.

Supplemental Figure III: Forest plot showing the effect sizes of published cIMT-associated SNPs in UK Biobank.

Supplemental Figure IV: Forest plot showing the effect sizes of novel cIMT-associated SNPs in UK Biobank.

Supplemental Table I: Comparison of GWAS significant SNPs for IMTmean on IMTmax

Supplemental Table II: Effects of previsouly reported IMT loci in the UKB GWAS

Supplemental Table III: Effects of GWAS-significant loci reported here in the CHARGE meta-analysis of IMT.

Supplemental Table IV: Comparison of GWAS significant SNPs for IMTmean in men and women

Supplemental Table V: Genetic correlations with IMTmean by sex

Supplemental Table VI: Previous associations with SNPs in GWAS-significant loci

Supplemental Table VII: Predicted functional, coding or loss of function variants

Supplemental Table VIII: EQTLS identified in GTEx

Supplemental Table IX: Baseline demographic characteristics of the individuals included in the analysis of ISH

Supplemental Table X: Baseline demographic characteristics of the individuals included in the analysis of stroke

Supplemental Table XI: Effect of IMT SNPs on ISH or stroke in UKB

Major Resources Table

Data & Code Availability

Description	Source / Repository	Persistent ID / URL
UK Biobank genetic and phenotypic data	UK Biobank	https://www.ukbiobank.ac.uk/
Analysis Code	Available upon request	NA

Supplemental Figure I

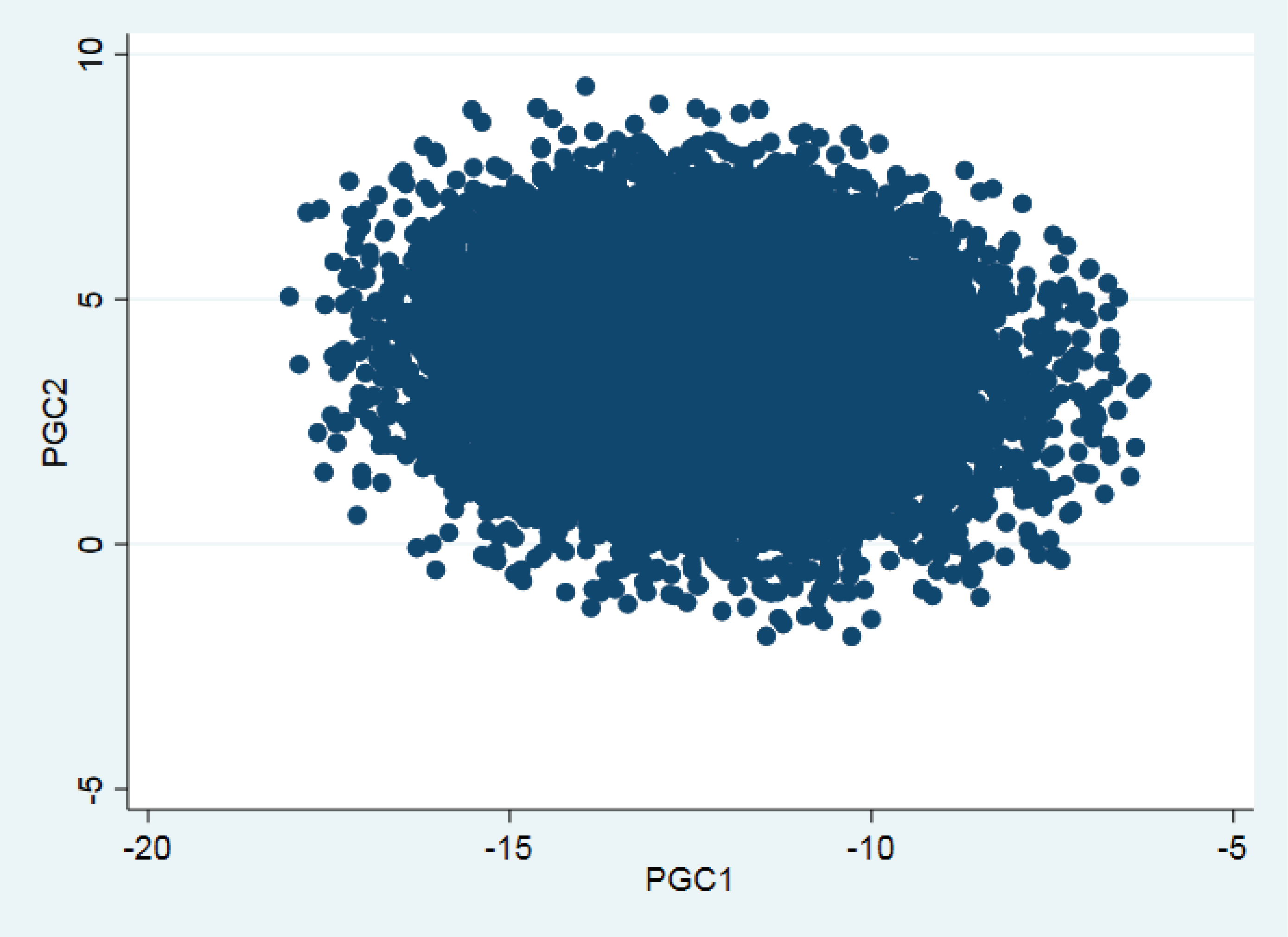

Supplemental Figure II

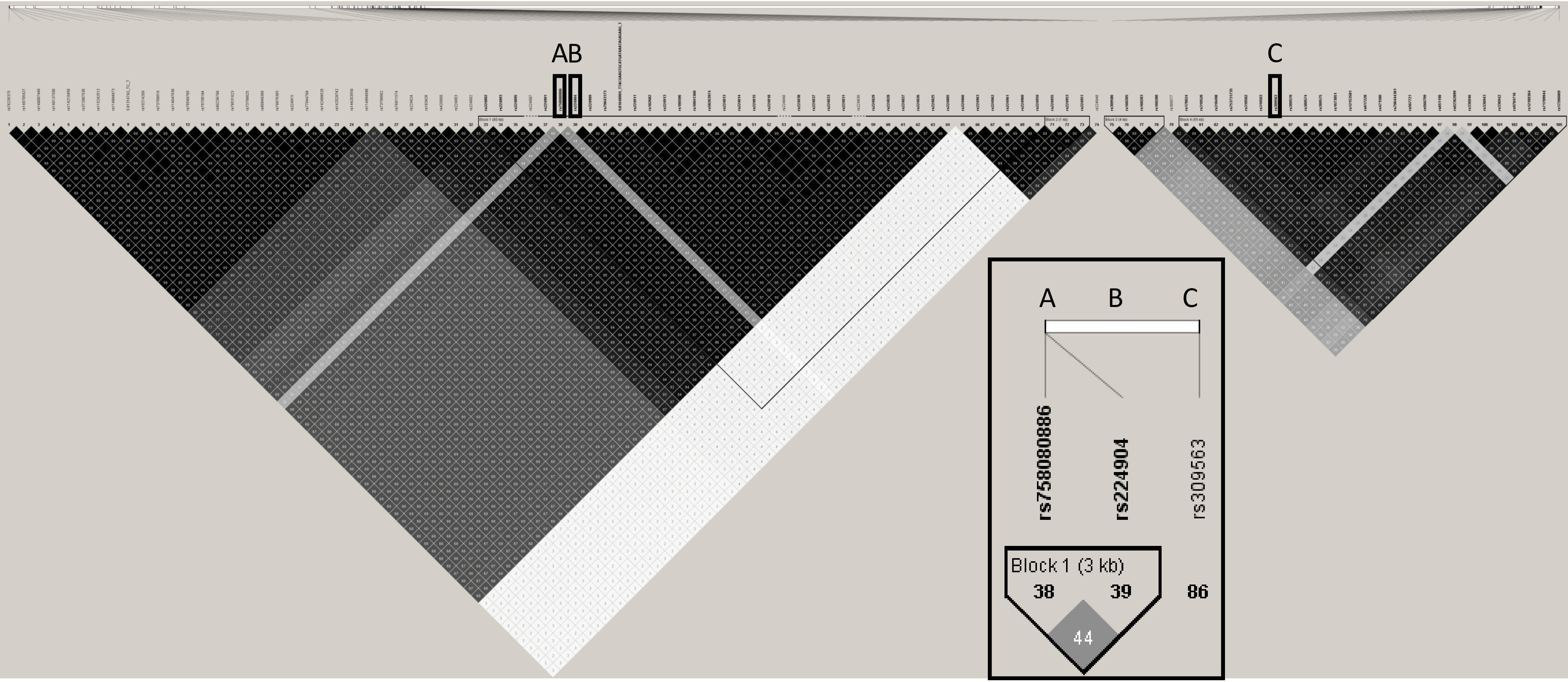

Supplemental Figure III

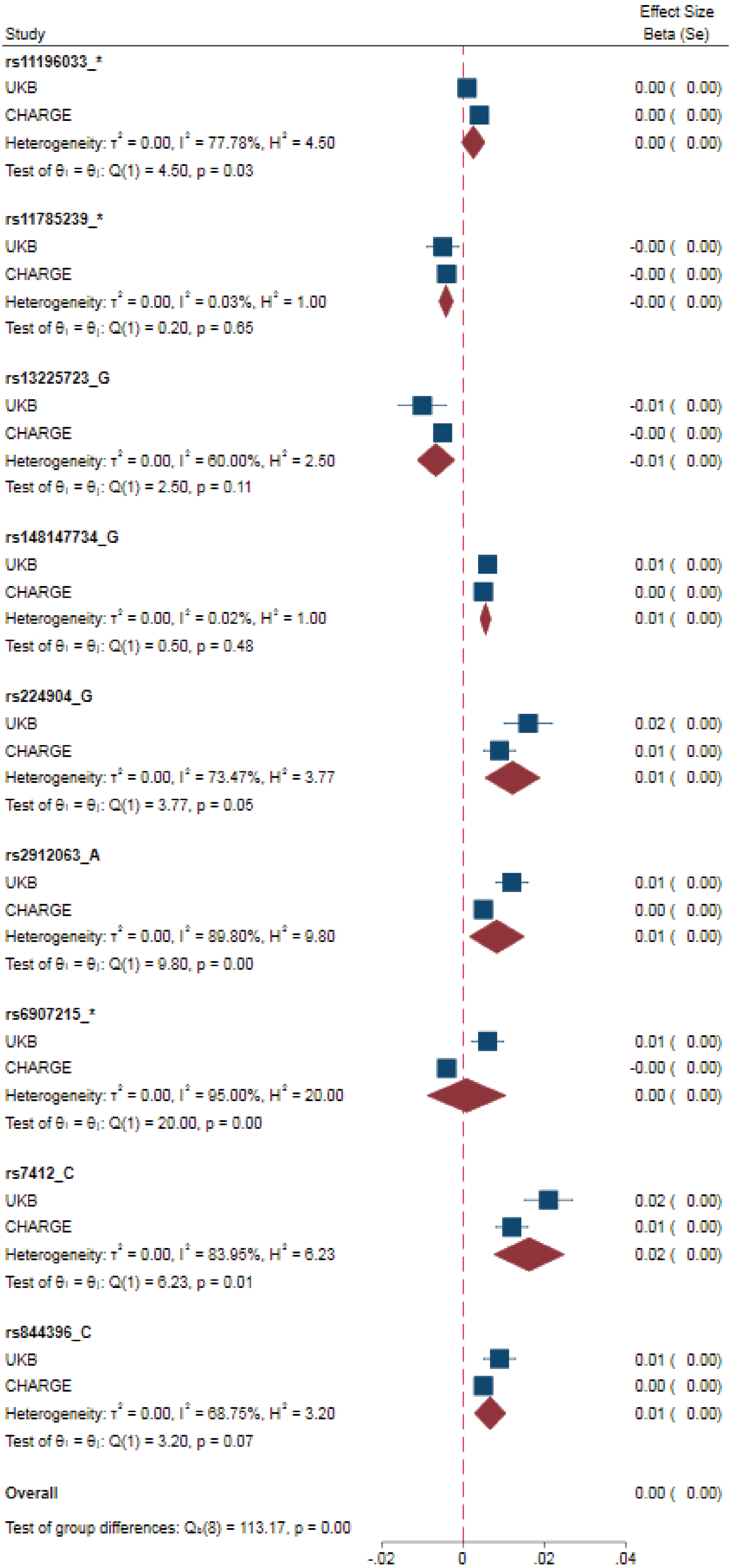

Supplemental Figure IV

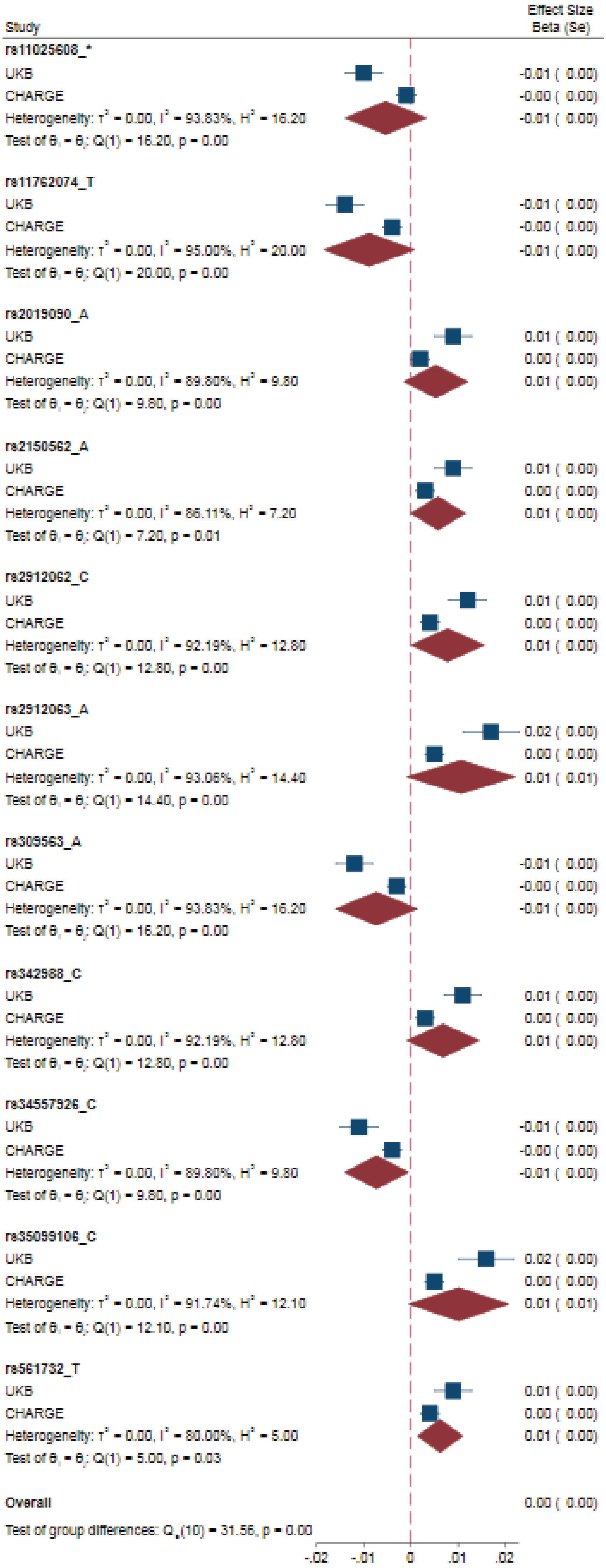

Supplementary Table I: Comparison of GWAS significant SNPs for IMTmean on IMTmax

						IMTmean			IMTmax			IMTmeanmax		
CHR	BP	SNP	A1	A0	A1freq	Beta	se	P	Beta	se	P	Beta	se	P
5	81616381	rs420800	G	A	0.95	-0.017	0.003	**4.30E-08**	-0.018	0.004	9.20E-06	-0.017	0.003	3.80E-07
5	81618075	rs224803	C	T	0.95	-0.017	0.003	**4.80E-08**	-0.018	0.004	1.00E-05	-0.017	0.003	4.40E-07
5	81634499	rs758080886	CA	C	0.15	0.013	0.002	**3.10E-08**	0.015	0.003	2.10E-07	0.011	0.002	1.60E-06
5	81683739	rs224805	A	G	0.05	0.017	0.003	**4.10E-08**	0.017	0.004	2.50E-05	0.016	0.003	8.60E-07
5	81699253	rs224960	A	G	0.52	0.008	0.001	**4.90E-08**	0.008	0.002	8.00E-06	0.007	0.001	4.40E-07
7	35423859	rs2541899	C	T	0.24	0.009	0.002	**2.20E-08**	0.009	0.002	1.70E-05	0.009	0.002	8.30E-08
7	35428513	rs12155459	A	T	0.77	-0.011	0.002	**2.40E-10**	-0.013	0.002	**2.80E-09**	-0.012	0.002	**3.20E-11**
7	35429465	rs11766731	G	T	0.77	-0.011	0.002	**1.60E-10**	-0.013	0.002	**1.90E-09**	-0.012	0.002	**2.20E-11**
7	35435835	rs2541905	T	C	0.23	0.011	0.002	**4.60E-10**	0.013	0.002	**2.50E-09**	0.011	0.002	**5.00E-11**
7	35436932	rs70981917	C	CT	0.43	0.008	0.001	**9.70E-09**	0.009	0.002	**2.70E-06**	0.008	0.001	**1.80E-08**
7	35438779	rs2013385	A	G	0.32	0.009	0.002	**5.10E-10**	0.011	0.002	**2.00E-08**	0.010	0.002	**3.30E-10**
7	35438831	rs741408	T	C	0.23	0.011	0.002	**3.80E-11**	0.013	0.002	**7.50E-10**	0.012	0.002	**6.30E-12**
7	35440055	rs2592856	C	T	0.23	0.011	0.002	**2.60E-11**	0.013	0.002	**5.50E-10**	0.012	0.002	**3.70E-12**
7	35440155	rs11762074	T	C	0.77	-0.011	0.002	**1.80E-11**	-0.014	0.002	**4.30E-10**	-0.012	0.002	**2.70E-12**
7	35446189	rs2592855	T	C	0.23	0.011	0.002	**2.40E-11**	0.013	0.002	**5.20E-10**	0.012	0.002	**4.30E-12**
7	35448018	rs2250212	A	G	0.32	0.010	0.002	**3.10E-10**	0.011	0.002	**1.20E-08**	0.010	0.002	**2.00E-10**
7	35449334	rs998652	A	G	0.23	0.011	0.002	**2.60E-11**	0.013	0.002	**5.70E-10**	0.012	0.002	**4.80E-12**
7	35451830	rs17765314	G	A	0.77	-0.011	0.002	**2.50E-11**	-0.013	0.002	**5.70E-10**	-0.012	0.002	**4.70E-12**
7	35451910	rs2592851	T	C	0.23	0.011	0.002	**2.50E-11**	0.013	0.002	**5.60E-10**	0.012	0.002	**4.50E-12**
7	35452092	rs1362655	G	A	0.23	0.011	0.002	**2.60E-11**	0.013	0.002	**5.90E-10**	0.012	0.002	**4.90E-12**
7	35455088	rs2592850	T	A	0.32	0.010	0.002	**2.80E-10**	0.011	0.002	**1.30E-08**	0.010	0.002	**2.10E-10**
7	35456691	rs2592849	A	T	0.32	0.010	0.002	**3.20E-10**	0.011	0.002	**1.40E-08**	0.010	0.002	**2.10E-10**
7	35457954	7:35457954_AT_A	AT	A	0.32	0.009	0.002	**5.50E-10**	0.012	0.002	**3.90E-09**	0.010	0.002	**1.70E-10**
7	35458137	rs140476889	A	AAAAC	0.32	0.010	0.002	**2.90E-10**	0.011	0.002	**1.20E-08**	0.010	0.002	**1.90E-10**
7	35458551	rs200832044	GT	G	0.32	0.009	0.002	**9.00E-10**	0.011	0.002	**3.20E-08**	0.010	0.002	**5.70E-10**
7	35458934	rs342976	T	C	0.32	0.009	0.002	**4.40E-10**	0.011	0.002	**1.90E-08**	0.010	0.002	**2.80E-10**

7	35459888	rs342977	G	A	0.23	0.011	0.002	**4.40E-11**	0.013	0.002	**1.10E-09**	0.012	0.002	**6.70E-12**
7	35460547	rs342979	C	T	0.32	0.009	0.002	**3.90E-10**	0.011	0.002	**1.60E-08**	0.010	0.002	**2.60E-10**
7	35461538	rs342981	G	A	0.32	0.010	0.002	**3.30E-10**	0.011	0.002	**1.60E-08**	0.010	0.002	**2.10E-10**
7	35461715	rs2551797	T	C	0.32	0.010	0.002	**3.30E-10**	0.011	0.002	**1.60E-08**	0.010	0.002	**2.10E-10**
7	35462145	rs342982	G	T	0.32	0.010	0.002	**3.20E-10**	0.011	0.002	**1.60E-08**	0.010	0.002	**2.00E-10**
7	35464620	rs34502079	A	AT	0.33	0.010	0.002	**8.30E-11**	0.012	0.002	**3.00E-09**	0.010	0.002	**2.50E-11**
7	35464784	rs342983	T	G	0.27	0.010	0.002	**1.70E-10**	0.012	0.002	**3.20E-08**	0.011	0.002	**7.70E-11**
7	35466464	rs342986	G	A	0.23	0.011	0.002	**3.90E-11**	0.013	0.002	**1.00E-09**	0.012	0.002	**5.00E-12**
7	35466658	rs35361607	A	AC	0.37	0.008	0.001	**2.80E-08**	0.009	0.002	1.80E-06	0.008	0.001	**1.70E-08**
7	35466755	rs342987	T	G	0.23	0.011	0.002	**3.70E-11**	0.013	0.002	**1.00E-09**	0.012	0.002	**4.80E-12**
7	35467026	rs342988	C	T	0.28	0.011	0.002	**8.90E-12**	0.013	0.002	**8.80E-10**	0.011	0.002	**1.30E-12**
7	35467677	7:35467677_TAA_T	TAA	T	0.24	0.011	0.002	**3.80E-11**	0.013	0.002	**1.00E-09**	0.012	0.002	**2.80E-12**
7	35467896	rs342989	A	G	0.23	0.011	0.002	**3.60E-11**	0.013	0.002	**9.80E-10**	0.012	0.002	**5.50E-12**
7	35468763	rs17697812	T	C	0.77	-0.011	0.002	**4.90E-11**	-0.013	0.002	**1.20E-09**	-0.012	0.002	**6.90E-12**
7	35469045	rs342990	G	T	0.38	0.008	0.001	**2.80E-08**	0.009	0.002	8.50E-07	0.009	0.002	**9.30E-09**
7	35469691	rs342991	G	T	0.32	0.009	0.002	**8.20E-10**	0.011	0.002	**2.60E-08**	0.010	0.002	**4.40E-10**
7	35469835	rs342992	C	T	0.23	0.011	0.002	**4.90E-11**	0.013	0.002	**1.20E-09**	0.012	0.002	**6.10E-12**
7	35469898	rs342993	G	A	0.32	0.009	0.002	**7.20E-10**	0.011	0.002	**2.50E-08**	0.010	0.002	**4.00E-10**
7	35471979	rs342996	G	C	0.33	0.009	0.002	**6.00E-10**	0.011	0.002	**2.10E-08**	0.010	0.002	**2.60E-10**
7	35474622	rs342997	T	C	0.32	0.009	0.002	**7.20E-10**	0.011	0.002	**2.60E-08**	0.010	0.002	**4.10E-10**
7	35475980	rs342998	T	C	0.32	0.009	0.002	**7.20E-10**	0.011	0.002	**2.60E-08**	0.010	0.002	**3.60E-10**
7	35477175	rs343000	C	T	0.23	0.011	0.002	**5.60E-11**	0.013	0.002	**1.50E-09**	0.012	0.002	**7.30E-12**
7	35478327	rs343001	C	G	0.32	0.009	0.002	**7.30E-10**	0.011	0.002	**2.60E-08**	0.010	0.002	**3.60E-10**
7	35478515	rs343002	T	C	0.23	0.011	0.002	**5.60E-11**	0.013	0.002	**1.50E-09**	0.012	0.002	**7.20E-12**
7	35479439	rs343003	C	T	0.23	0.011	0.002	**5.90E-11**	0.013	0.002	**1.50E-09**	0.012	0.002	**7.50E-12**
7	35481642	rs343004	C	A	0.32	0.009	0.002	**9.00E-10**	0.011	0.002	**3.00E-08**	0.010	0.002	**4.10E-10**
7	35482940	rs343005	G	T	0.32	0.009	0.002	**8.70E-10**	0.011	0.002	**2.60E-08**	0.010	0.002	**3.80E-10**
7	35483018	rs343006	C	A	0.32	0.009	0.002	**9.10E-10**	0.011	0.002	**2.60E-08**	0.010	0.002	**4.00E-10**
7	35483055	rs343007	G	T	0.32	0.009	0.002	**8.00E-10**	0.011	0.002	**2.60E-08**	0.010	0.002	**3.70E-10**
7	35485648	rs343010	C	A	0.32	0.009	0.002	**7.50E-10**	0.011	0.002	**2.60E-08**	0.010	0.002	**3.40E-10**

7	35487040	7:35487040_CT_C	CT	C	0.29	0.011	0.002	**3.20E-09**	0.013	0.002	**1.60E-08**	0.011	0.002	**5.30E-09**
7	35488349	rs343011	T	G	0.23	0.011	0.002	**5.50E-11**	0.013	0.002	**1.50E-09**	0.012	0.002	**6.80E-12**
7	35488769	rs343012	C	A	0.32	0.009	0.002	**7.40E-10**	0.011	0.002	**2.70E-08**	0.010	0.002	**3.40E-10**
7	35491532	rs343016	G	C	0.32	0.009	0.002	**8.00E-10**	0.011	0.002	**2.60E-08**	0.010	0.002	**3.60E-10**
7	35492168	rs343019	G	C	0.41	0.009	0.001	**7.10E-10**	0.010	0.002	1.60E-07	0.009	0.001	**3.00E-10**
7	35493178	rs343020	T	C	0.32	0.009	0.002	**8.20E-10**	0.011	0.002	**2.40E-08**	0.010	0.002	**3.70E-10**
7	35493309	rs343021	T	C	0.32	0.009	0.002	**8.10E-10**	0.011	0.002	**2.40E-08**	0.010	0.002	**3.70E-10**
7	35493603	rs343022	C	T	0.32	0.009	0.002	**8.70E-10**	0.011	0.002	**2.20E-08**	0.010	0.002	**3.50E-10**
7	35494013	rs343024	C	G	0.41	0.009	0.001	**5.80E-10**	0.010	0.002	1.20E-07	0.009	0.001	**2.40E-10**
7	35494243	rs777980	G	A	0.32	0.009	0.002	**6.10E-10**	0.011	0.002	**1.70E-08**	0.010	0.002	**2.40E-10**
7	35494556	rs702843	G	C	0.41	0.009	0.001	**3.90E-10**	0.010	0.002	**7.30E-08**	0.009	0.001	**1.40E-10**
7	35495599	rs343025	T	A	0.37	0.009	0.001	**5.00E-09**	0.010	0.002	2.80E-07	0.009	0.001	**3.50E-09**
7	35496678	rs343026	T	G	0.32	0.009	0.002	**5.20E-10**	0.011	0.002	**1.50E-08**	0.010	0.002	**2.40E-10**
7	35497055	rs35099106	C	CT	0.23	0.011	0.002	**3.70E-11**	0.013	0.002	**8.70E-10**	0.012	0.002	**4.10E-12**
7	35498200	rs343029	A	G	0.23	0.011	0.002	**3.50E-11**	0.013	0.002	**8.90E-10**	0.012	0.002	**4.50E-12**
7	35500493	rs343031	G	A	0.32	0.009	0.002	**5.00E-10**	0.011	0.002	**1.50E-08**	0.010	0.002	**2.20E-10**
7	35501883	rs343034	C	T	0.37	0.009	0.001	**5.20E-09**	0.010	0.002	2.70E-07	0.009	0.002	**3.50E-09**
7	35503445	rs343036	G	C	0.23	0.011	0.002	**3.50E-11**	0.013	0.002	**8.70E-10**	0.012	0.002	**5.30E-12**
7	35503600	rs343037	G	T	0.23	0.011	0.002	**3.60E-11**	0.013	0.002	**8.70E-10**	0.012	0.002	**5.30E-12**
7	35503696	rs343038	G	C	0.32	0.009	0.002	**4.70E-10**	0.011	0.002	**1.40E-08**	0.010	0.002	**2.40E-10**
7	35505204	rs343039	C	T	0.23	0.011	0.002	**3.90E-11**	0.013	0.002	**7.90E-10**	0.012	0.002	**5.20E-12**
7	35508859	rs343044	A	G	0.20	0.010	0.002	**3.60E-08**	0.012	0.002	4.30E-07	0.011	0.002	**5.20E-09**
7	35509024	rs343045	C	A	0.20	0.010	0.002	**3.60E-08**	0.012	0.002	4.40E-07	0.011	0.002	**5.00E-09**
7	35515295	rs343048	G	A	0.20	0.010	0.002	**3.30E-08**	0.012	0.002	4.20E-07	0.011	0.002	**4.30E-09**
7	35519519	rs343052	G	T	0.20	0.010	0.002	**3.50E-08**	0.012	0.002	4.20E-07	0.011	0.002	**4.70E-09**
7	35522553	rs343053	A	G	0.17	0.011	0.002	**4.30E-09**	0.012	0.002	4.60E-07	0.011	0.002	**3.90E-09**
7	35523883	rs343055	G	A	0.17	0.011	0.002	**3.50E-09**	0.013	0.002	2.90E-07	0.012	0.002	**2.70E-09**
7	35526022	rs1646989	T	G	0.20	0.010	0.002	**1.50E-08**	0.012	0.002	1.60E-07	0.011	0.002	**2.00E-09**
7	35526024	rs1646988	T	A	0.20	0.010	0.002	**1.50E-08**	0.012	0.002	1.60E-07	0.011	0.002	**2.10E-09**
7	35526390	rs343056	A	G	0.20	0.010	0.002	**7.00E-09**	0.012	0.002	1.30E-07	0.011	0.002	**9.70E-10**

7	35528567	rs11766156	G	C	0.80	-0.010	0.002	**7.40E-09**	-0.012	0.002	1.20E-07	-0.011	0.002	**8.90E-10**
7	35532991	rs73087411	C	T	0.82	-0.010	0.002	**4.60E-08**	-0.011	0.002	2.80E-06	-0.011	0.002	**7.60E-09**
8	6470650	rs3020257	C	A	0.76	0.010	0.002	**6.60E-10**	0.009	0.002	8.10E-05	0.009	0.002	**3.30E-08**
8	6471126	rs2980664	G	A	0.76	0.011	0.002	**2.50E-10**	0.009	0.002	2.60E-05	0.010	0.002	**1.00E-08**
8	6471182	rs3020258	C	T	0.78	0.010	0.002	**2.00E-09**	0.009	0.002	9.60E-05	0.009	0.002	9.90E-08
8	6471995	rs2959802	G	A	0.42	-0.008	0.001	**3.30E-08**	-0.007	0.002	9.20E-05	-0.008	0.001	2.10E-07
8	6472105	rs2959801	G	A	0.69	0.010	0.002	**2.00E-10**	0.009	0.002	9.10E-06	0.010	0.002	**4.80E-10**
8	6472603	rs11137038	G	A	0.64	0.010	0.001	**6.30E-11**	0.008	0.002	2.00E-05	0.009	0.002	**3.90E-09**
8	6472721	rs12543564	T	C	0.67	0.009	0.002	**1.30E-08**	0.009	0.002	1.70E-05	0.009	0.002	**3.70E-08**
8	6472795	rs3020259	C	A	0.65	0.009	0.001	**2.30E-10**	0.008	0.002	2.10E-05	0.009	0.002	**2.20E-08**
8	6473118	rs11998083	C	T	0.64	0.010	0.001	**4.50E-11**	0.008	0.002	1.30E-05	0.009	0.002	**4.20E-09**
8	6473356	rs1256	C	G	0.70	0.010	0.002	**1.80E-11**	0.010	0.002	1.20E-06	0.010	0.002	**1.10E-10**
8	6473715	rs2959799	A	G	0.63	0.010	0.001	**1.10E-10**	0.008	0.002	1.80E-05	0.009	0.002	**5.90E-09**
8	6473746	rs2959798	A	G	0.63	0.009	0.001	**1.20E-10**	0.008	0.002	1.80E-05	0.009	0.002	**6.20E-09**
8	6473758	rs2454517	A	G	0.70	0.010	0.002	**4.30E-11**	0.010	0.002	2.40E-06	0.010	0.002	**2.70E-10**
8	6474136	rs2936501	A	G	0.63	0.009	0.001	**1.60E-10**	0.008	0.002	1.70E-05	0.009	0.002	**7.90E-09**
8	6475113	rs2515557	G	A	0.70	0.009	0.002	**3.90E-08**	0.008	0.002	6.60E-05	0.008	0.002	3.30E-07
8	6475489	rs2980662	G	C	0.69	0.009	0.002	**2.60E-09**	0.009	0.002	1.90E-05	0.009	0.002	5.40E-08
8	6476626	rs7005662	C	T	0.72	0.010	0.002	**1.50E-10**	0.010	0.002	2.50E-06	0.010	0.002	**1.90E-09**
8	6478481	rs2253560	G	A	0.72	0.010	0.002	**7.80E-11**	0.009	0.002	4.20E-06	0.010	0.002	**2.90E-09**
8	6478960	rs11137040	C	G	0.71	0.010	0.002	**7.90E-10**	0.009	0.002	2.70E-05	0.009	0.002	**2.10E-08**
8	6479178	rs2912016	C	A	0.65	0.009	0.001	**6.70E-10**	0.008	0.002	4.70E-05	0.008	0.002	1.30E-07
8	6479282	rs11137041	G	T	0.65	0.009	0.001	**2.20E-09**	0.008	0.002	7.30E-05	0.008	0.002	1.60E-07
8	6479432	rs2515559	A	G	0.72	0.010	0.002	**5.10E-10**	0.009	0.002	8.40E-06	0.009	0.002	**1.50E-08**
8	6479497	rs2011423	G	A	0.67	0.009	0.002	**1.40E-09**	0.008	0.002	4.10E-05	0.008	0.002	2.20E-07
8	6483059	rs201338894	ATAAAT	A	0.73	0.011	0.002	**9.30E-12**	0.010	0.002	1.70E-06	0.010	0.002	**6.60E-10**
8	6483482	rs2912057	C	T	0.60	0.008	0.001	**2.60E-08**	0.008	0.002	4.10E-05	0.007	0.001	1.30E-06
8	6484505	8:6484505_CA_C	CA	C	0.72	0.011	0.002	**3.10E-12**	0.010	0.002	9.40E-07	0.010	0.002	**2.30E-10**
8	6484510	rs2454524	A	C	0.72	0.011	0.002	**3.10E-12**	0.010	0.002	9.50E-07	0.010	0.002	**2.30E-10**
8	6484530	rs2246801	C	T	0.72	0.011	0.002	**4.50E-12**	0.010	0.002	1.10E-06	0.010	0.002	**3.80E-10**

8	6484635	rs2278147	C	A	0.71	0.011	0.002	**4.10E-13**	0.010	0.002	6.10E-07	0.010	0.002	**4.80E-11**
8	6484982	rs2448611	C	T	0.70	0.010	0.002	**1.50E-10**	0.010	0.002	1.30E-06	0.009	0.002	**4.40E-09**
8	6485295	rs2912062	C	G	0.70	0.011	0.002	**2.70E-13**	0.010	0.002	2.70E-07	0.011	0.002	**3.30E-11**
8	6486033	rs2912063	A	G	0.71	0.011	0.002	**4.20E-13**	0.010	0.002	4.80E-07	0.011	0.002	**4.40E-11**
8	6487449	rs3020263	G	A	0.72	0.011	0.002	**4.40E-12**	0.010	0.002	1.00E-06	0.010	0.002	**2.40E-10**
8	6487727	rs2936511	C	G	0.63	0.008	0.001	**2.20E-08**	0.007	0.002	5.30E-04	0.007	0.001	2.60E-06
8	6488317	rs569123053	A	C	0.80	0.011	0.002	**2.70E-09**	0.010	0.002	6.50E-05	0.010	0.002	2.60E-07
8	6488505	rs2911968	T	C	0.62	0.008	0.001	**2.30E-08**	0.007	0.002	4.20E-04	0.007	0.001	2.40E-06
8	6488577	rs3020264	G	A	0.72	0.011	0.002	**5.80E-12**	0.010	0.002	1.40E-06	0.010	0.002	**4.30E-10**
8	6488710	rs2912064	C	T	0.65	0.009	0.001	**2.60E-10**	0.008	0.002	1.10E-04	0.008	0.002	8.20E-08
8	6488787	rs1974946	G	A	0.72	0.011	0.002	**5.70E-12**	0.010	0.002	1.60E-06	0.010	0.002	**4.00E-10**
8	6488921	rs3020265	G	A	0.63	0.008	0.001	**1.60E-08**	0.007	0.002	3.30E-04	0.007	0.001	1.50E-06
8	6488990	8:6488990_CA_C	CA	C	0.62	0.008	0.001	**2.40E-08**	0.007	0.002	3.60E-04	0.007	0.002	4.00E-06
8	6490200	rs2433143	C	T	0.73	0.010	0.002	**6.50E-11**	0.009	0.002	3.40E-05	0.010	0.002	**1.20E-09**
8	6490544	8:6490544_TA_T	TA	T	0.70	0.010	0.002	**2.00E-11**	0.008	0.002	3.90E-05	0.010	0.002	**9.60E-10**
8	6490975	rs2515561	G	A	0.66	0.009	0.001	**6.70E-10**	0.007	0.002	2.60E-04	0.008	0.002	**2.60E-08**
8	6491181	rs1834451	T	C	0.61	0.009	0.001	**1.60E-09**	0.007	0.002	2.70E-04	0.008	0.001	7.10E-08
8	6492014	8:6492014_CTG_C	CTG	C	0.67	0.008	0.002	**1.90E-08**	0.006	0.002	9.60E-04	0.008	0.002	7.30E-07
8	6492391	rs2980655	A	T	0.67	0.009	0.002	**6.70E-09**	0.007	0.002	4.70E-04	0.008	0.002	3.10E-07
8	6492498	rs3020266	G	A	0.67	0.009	0.002	**8.20E-09**	0.007	0.002	5.20E-04	0.008	0.002	3.70E-07
8	6492792	8:6492792_AC_A	AC	A	0.66	0.009	0.002	**1.10E-08**	0.007	0.002	5.00E-04	0.008	0.002	2.30E-07
8	6493910	rs2042670	A	C	0.66	0.008	0.001	**1.40E-08**	0.006	0.002	1.80E-03	0.008	0.002	5.60E-07
8	6494850	rs2515563	G	C	0.69	0.010	0.002	**2.50E-10**	0.008	0.002	9.50E-05	0.009	0.002	**1.20E-08**
8	124579985	rs6470156	G	A	0.73	-0.011	0.002	**5.30E-13**	-0.009	0.002	2.10E-05	-0.010	0.002	**1.90E-09**
8	124594184	rs13270370	G	A	0.55	-0.008	0.001	**2.40E-08**	-0.006	0.002	2.60E-03	-0.007	0.001	4.20E-06
8	124594923	rs11776999	G	A	0.80	-0.010	0.002	**3.50E-08**	-0.008	0.002	5.40E-04	-0.009	0.002	1.50E-06
8	124599786	rs13251106	C	T	0.80	-0.010	0.002	**3.60E-08**	-0.008	0.002	5.60E-04	-0.009	0.002	1.50E-06
8	124601535	rs12547541	G	A	0.80	-0.010	0.002	**3.70E-08**	-0.008	0.002	5.50E-04	-0.009	0.002	1.60E-06
8	124602054	rs35534969	A	C	0.55	-0.008	0.001	**2.20E-08**	-0.006	0.002	2.40E-03	-0.007	0.001	5.50E-06
8	124607159	rs34557926	C	T	0.64	-0.011	0.001	**1.40E-13**	-0.008	0.002	2.60E-05	-0.009	0.002	**1.00E-09**

8	124608162	rs2891781	C	T	0.64	-0.011	0.001	**2.70E-13**	-0.008	0.002	3.90E-05	-0.009	0.002	**1.20E-09**
8	124608614	rs7006122	T	G	0.64	-0.011	0.001	**3.10E-13**	-0.008	0.002	4.50E-05	-0.009	0.002	**1.40E-09**
10	30138169	rs7912501	A	G	0.30	0.009	0.002	**2.40E-08**	0.007	0.002	1.20E-03	0.008	0.002	1.10E-07
10	30138411	rs6481643	T	C	0.30	0.009	0.002	**2.20E-08**	0.007	0.002	1.20E-03	0.008	0.002	1.00E-07
10	30144471	rs10763762	T	C	0.30	0.008	0.002	**4.80E-08**	0.006	0.002	1.60E-03	0.008	0.002	2.10E-07
10	30161168	rs1929257	A	G	0.31	0.009	0.002	**6.00E-09**	0.007	0.002	5.70E-04	0.009	0.002	**1.80E-08**
10	30162423	rs2150562	A	G	0.31	0.009	0.002	**8.70E-09**	0.007	0.002	5.60E-04	0.009	0.002	**1.70E-08**
10	30163133	rs777393148	TC	T	0.31	0.008	0.002	**3.30E-08**	0.007	0.002	1.00E-03	0.008	0.002	6.20E-08
10	30165983	rs7096778	T	C	0.41	0.008	0.001	**1.60E-08**	0.007	0.002	1.40E-04	0.008	0.001	1.60E-07
10	30166035	rs7078837	C	A	0.41	0.008	0.001	**1.40E-08**	0.007	0.002	1.10E-04	0.008	0.001	1.30E-07
10	30166547	rs10826719	G	A	0.41	0.008	0.001	**1.70E-08**	0.007	0.002	1.30E-04	0.008	0.001	1.80E-07
10	30167009	rs1571759	T	C	0.41	0.008	0.001	**1.30E-08**	0.007	0.002	1.20E-04	0.008	0.001	1.50E-07
10	30167754	rs10740811	G	A	0.41	0.008	0.001	**1.10E-08**	0.007	0.002	8.50E-05	0.008	0.001	1.20E-07
10	30168031	rs2369339	T	C	0.32	0.009	0.002	**5.10E-09**	0.007	0.002	2.60E-04	0.009	0.002	**2.00E-08**
10	30168699	rs2202	C	T	0.32	0.009	0.002	**8.80E-09**	0.007	0.002	2.80E-04	0.009	0.002	**2.80E-08**
10	30169653	rs10763764	A	T	0.42	0.008	0.001	**1.10E-08**	0.007	0.002	8.60E-05	0.008	0.001	1.00E-07
10	30170487	rs914279	T	G	0.42	0.008	0.001	**6.80E-09**	0.007	0.002	7.00E-05	0.008	0.001	5.80E-08
11	103660567	rs974819	T	C	0.29	0.010	0.002	**5.60E-10**	0.010	0.002	3.70E-07	0.010	0.002	**8.30E-10**
11	103668962	rs2019090	A	T	0.29	0.010	0.002	**3.70E-10**	0.010	0.002	3.30E-07	0.010	0.002	**6.40E-10**
11	103669291	rs796784254	T	TTATTGAA	0.29	0.010	0.002	**5.80E-10**	0.010	0.002	4.20E-07	0.010	0.002	**7.80E-10**
11	103673277	rs2128739	A	C	0.28	0.009	0.002	**2.20E-09**	0.010	0.002	1.10E-06	0.009	0.002	**3.60E-09**
11	103673294	rs2839812	T	A	0.28	0.009	0.002	**2.20E-09**	0.010	0.002	1.10E-06	0.009	0.002	**3.60E-09**
11	103693627	rs11226029	G	A	0.29	0.009	0.002	**1.10E-08**	0.009	0.002	3.30E-06	0.009	0.002	**3.10E-08**
11	103696851	rs1384705	C	T	0.29	0.009	0.002	**1.30E-08**	0.009	0.002	3.20E-06	0.009	0.002	**3.60E-08**
13	111049623	rs9515203	T	C	0.74	-0.009	0.002	**1.10E-08**	-0.009	0.002	1.20E-05	-0.009	0.002	1.80E-07
16	88966667	rs844396	C	T	0.68	0.008	0.002	**4.50E-08**	0.009	0.002	1.40E-05	0.008	0.002	2.30E-07
16	88968540	rs564669	T	C	0.62	0.008	0.001	**4.30E-08**	0.008	0.002	2.70E-05	0.008	0.001	2.90E-07
16	88969969	rs865102	A	G	0.65	0.008	0.001	**9.10E-09**	0.008	0.002	1.30E-05	0.008	0.002	1.30E-07
16	88970040	rs4995274	T	C	0.65	0.008	0.001	**3.20E-08**	0.008	0.002	2.90E-05	0.008	0.002	3.50E-07

16	88970776	rs475796	C	G	0.65	0.008	0.001	**1.00E-08**	0.008	0.002	1.40E-05	0.008	0.002	1.40E-07
16	88970818	rs561795	A	G	0.65	0.008	0.001	**1.30E-08**	0.008	0.002	1.30E-05	0.008	0.002	1.50E-07
16	88972554	rs488251	G	A	0.68	0.008	0.002	**3.30E-08**	0.008	0.002	4.00E-05	0.008	0.002	4.90E-07
16	88974516	rs548591	A	G	0.62	0.008	0.001	**1.80E-08**	0.008	0.002	1.60E-05	0.008	0.001	2.70E-07
16	88974860	rs533406	A	G	0.62	0.008	0.001	**2.70E-08**	0.008	0.002	2.40E-05	0.008	0.001	3.30E-07
16	88975447	rs8058234	C	G	0.62	0.008	0.001	**3.20E-08**	0.008	0.002	4.10E-05	0.008	0.001	4.40E-07
16	88975910	rs7405380	G	C	0.62	0.008	0.001	**2.20E-08**	0.008	0.002	3.80E-05	0.008	0.001	3.40E-07
16	88975954	rs7404039	A	G	0.62	0.008	0.001	**2.70E-08**	0.008	0.002	5.20E-05	0.008	0.001	4.30E-07
16	88976477	rs1673931	T	C	0.62	0.008	0.001	**2.30E-08**	0.008	0.002	3.50E-05	0.008	0.001	3.80E-07
16	88976968	rs529900	G	C	0.62	0.008	0.001	**2.50E-08**	0.008	0.002	3.90E-05	0.008	0.001	3.50E-07
16	88982789	rs12444726	G	T	0.63	0.008	0.001	**4.70E-08**	0.007	0.002	2.90E-04	0.007	0.001	2.30E-06
16	88987861	rs1673932	T	C	0.63	0.008	0.001	**3.50E-08**	0.008	0.002	5.30E-05	0.007	0.001	8.60E-07
16	88988989	rs561732	T	C	0.65	0.008	0.001	**4.70E-08**	0.008	0.002	3.80E-05	0.007	0.002	7.10E-07
19	41188985	rs113228202	G	A	0.99	-0.036	0.007	**3.40E-08**	-0.034	0.008	6.70E-05	-0.036	0.007	8.40E-08
19	41333152	rs111689747	G	A	0.99	-0.040	0.006	**7.10E-10**	-0.044	0.008	2.20E-07	-0.039	0.007	**4.40E-09**
19	41371458	rs111240159	C	T	0.99	-0.037	0.006	**4.00E-09**	-0.041	0.008	3.60E-07	-0.037	0.006	**8.30E-09**
19	45337737	rs11667253	C	T	0.30	-0.009	0.002	**2.70E-08**	-0.009	0.002	1.10E-05	-0.009	0.002	**2.60E-08**
19	45338220	rs4369782	A	G	0.30	-0.009	0.002	**2.90E-08**	-0.009	0.002	9.60E-06	-0.009	0.002	**2.80E-08**
19	45338493	rs4605275	T	C	0.30	-0.009	0.002	**3.40E-08**	-0.009	0.002	9.20E-06	-0.009	0.002	**2.70E-08**
19	45341948	rs10407439	A	G	0.30	-0.009	0.002	**2.30E-08**	-0.009	0.002	9.90E-06	-0.009	0.002	**2.40E-08**
19	45342241	rs4239533	A	G	0.30	-0.009	0.002	**1.80E-08**	-0.009	0.002	8.50E-06	-0.009	0.002	**2.10E-08**
19	45397229	rs1160983	G	A	0.97	0.023	0.004	**4.70E-08**	0.030	0.005	**1.70E-08**	0.026	0.004	**3.90E-10**
19	45412079	rs7412	C	T	0.92	0.021	0.003	**2.10E-15**	0.025	0.003	**1.00E-13**	0.022	0.003	**1.40E-16**
19	45413233	rs1065853	G	T	0.92	0.021	0.003	**1.60E-15**	0.026	0.003	**6.90E-14**	0.023	0.003	**7.80E-17**
19	45414399	rs72654473	C	A	0.90	0.013	0.002	**2.40E-08**	0.017	0.003	**8.70E-09**	0.014	0.002	**1.60E-09**
19	45415640	rs445925	G	A	0.89	0.013	0.002	**3.20E-08**	0.017	0.003	**1.30E-08**	0.014	0.002	**2.50E-09**
19	45416831	rs390082	T	G	0.89	0.013	0.002	**4.10E-08**	0.017	0.003	**2.00E-08**	0.014	0.002	**3.50E-09**
19	45425178	rs190712692	G	A	0.95	0.023	0.003	**1.30E-12**	0.028	0.004	**7.80E-11**	0.025	0.003	**4.00E-14**
19	45426792	rs141622900	G	A	0.95	0.023	0.003	**1.90E-12**	0.026	0.004	**4.00E-10**	0.025	0.003	**7.50E-14**

Supplemental Table II: Effects of previously reported IMT loci in the UKB GWAS

Franceshini							UKB						
SNP	Chr	Nearest Coding Gene	Alleles Effect/Other	Effect allele frequency	Beta (SE)	p-value	Alleles Effect/Other	A1freq	IMTmean beta	IMTmean P	IMTmeanmax beta	IMTmeanmax P	consistent
rs201648240	1		D/I	0.83	-0.0062 (0.0011)	3.6×10^{-9}							
rs224904	5	*ATP6AP1L*	C/G	0.95	-0.0088 (0.0016)	5.1×10^{-8}	G/C	0.05	0.0163	7.20E-07	0.0153	2.10E-06	y
rs6907215	6	*AIG1*	T/C	0.6	-0.0040 (0.0007)	4.7×10^{-8}	C/T	0.40	0.0060	6.80E-05	0.0060	4.80E-05	Y
rs13225723	7	*PIK3CG*	A/G	0.22	0.0052 (0.0009)	3.2×10^{-9}	G/A	0.89	-0.0097	0.0004	-0.0078	0.0031	y
rs2912063	8	*MCPH1*	A/G	0.71	0.0045 (0.0008)	8.9×10^{-9}	A/G	0.71	0.0122	**8.3E-14**	0.0105	**4.40E-11**	y
rs11785239	8	*SGK223*	T/C	0.65	-0.0043 (0.0008)	8.7×10^{-9}	C/T	0.37	-0.0045	0.0033	-0.0057	0.0002	n
rs11196033	10	*VTI1A*	A/C	0.48	0.0042 (0.0008)	3.9×10^{-8}	C/A	0.49	0.0011	0.4500	0.0007	0.6400	n
rs844396	16	*CBFA2T3*	T/C	0.3	-0.0051 (0.0009)	6.0×10^{-9}	C/T	0.68	0.0086	**4.80E-08**	0.0080	2.30E-07	y
rs148147734*	8	*ZHX2*	−/G	0.54	0.0050 (0.0007)	3×10^{-11}	G/A	0.54	0.0015	3.70E-05	0.0056	0.0001	y
rs200482500	8	*PINX1*	−/GTACC	0.52	0.0056 (0.0008)	7×10^{-12}							
rs7412	19	*APOE*	T/C	0.08	−0.0119 (0.0015)	1×10^{-14}	C/T	0.92	0.0214	**7.1E-15**	0.0224	**1.40E-16**	y

Where: *Published cIMT SNP in LD with our most significant SNP: rs11781551 (r2 = 0.95 with rs148147734)

Supplemental Table III: Effects of GWAS-significant loci reported here in the CHARGE meta-analysis of IMT.

			UKB						Franceshini et al						
Analysis	SNP	CHR	A1	A1 freq	IMTmean beta	IMTmean P	IMTmeanmax beta	IMTmeanmax P	A1	A1 Freq	Beta	Meta P	Het ISq	Het PVal	Consistent
IMTmean sex combined	rs758080886	5	CA	0.15	0.013	3.10E-08	0.011	1.60E-06							
	rs342988*,§	7	C	0.28	0.011	3.00E-11	0.011	1.30E-12	T	0.75	-0.003	1.53E-04	7.4	0.3533	y
	rs2912062†	8	C	0.71	0.012	6.70E-14	0.011	3.30E-11	C	0.70	0.004	5.53E-08	2.6	0.4260	y
	rs34557926§	8	C	0.64	-0.011	2.90E-12	-0.009	1.00E-09	T	0.38	0.004	3.45E-06	0	0.7801	y
	rs2019090§	11	A	0.29	0.009	8.20E-09	0.010	6.40E-10	A	0.28	0.002	0.0046	25.3	0.1048	y
	rs561732	16	T	0.65	0.009	3.60E-09	0.007	7.10E-07	T	0.67	0.004	1.95E-06	56.9	**0.0012**	y
	rs111689747§	19	G	0.99	-0.044	1.20E-10	-0.039	4.40E-09							
	rs1065853‡	19	G	0.92	0.021	6.20E-15	0.023	7.80E-17							
IMTmax sex combined	rs11762074*	7	T	0.77	-0.014	4.30E-10	-0.012	2.70E-12	T	0.79	-0.004	5.94E-07	27.6	0.0829	y
	rs11025608§	11	G	0.44	-0.010	3.20E-08	-0.007	6.50E-07	T	0.57	-0.001	0.1911	32.1	0.0566	N
	rs1065853‡	19	G	0.92	0.026	6.90E-14	0.023	7.80E-17							
IMTmeanmax sex combined	rs2150562||	10	A	0.31	0.008	1.50E-06	0.009	1.70E-08	A	0.34	0.003	0.0003	0	0.4714	Y
IMTmean Men	rs35099106*	7	C	0.24	0.016	5.20E-09	0.012	4.10E-12	C	0.21	0.005	3.65E-07	37.5	**0.0290**	y
	rs2912063†	8	A	0.71	0.017	2.60E-11	0.011	4.40E-11	A	0.71	0.005	**8.96E-09**	9	0.3292	y
	rs1065853‡	19	G	0.92	0.027	4.00E-10	0.023	7.80E-17							
IMTmean Women	rs309563§	5	A	0.66	-0.012	8.90E-10	-0.007	5.40E-06	A	0.66	-0.003	1.10E-05	13.5	0.2591	y

Where: *, LD between rs342988, rs11762074 and rs35099106 suggests that these SNPs repesent 1 signal; †, LD between rs2912063 and rs2912063 indicate that these SNPs are proxies; ‡, consistent lead SNP convincingly demonstrates that the same locus is being identified; §, novel locus.

Supplemental Table IV: Comparison of GWAS significant SNPs for IMTmean in men and women

					women				men				Sex difference	
CHR	BP	SNP	ALLELE1	ALLELE0	A1F	BETA	SE	P	A1F	BETA	SE	P	i2	P
5	82870033	5:82870033_TC_T	TC	T	0.65	-0.011	0.002	3.50E-08	0.66	-0.002	0.002	4.80E-01		
5	82871546	rs309581	T	C	0.65	-0.011	0.002	2.00E-08	0.66	-0.002	0.002	4.90E-01	0.88	0.0033
5	82871657	rs309580	A	T	0.65	-0.011	0.002	2.50E-08	0.66	-0.002	0.002	4.40E-01	0.88	0.0042
5	82873262	rs309579	G	T	0.65	-0.011	0.002	2.20E-08	0.66	-0.002	0.002	4.00E-01	0.87	0.0048
5	82874549	rs309578	G	A	0.65	-0.011	0.002	2.50E-08	0.66	-0.002	0.002	4.40E-01	0.88	0.0042
5	82874942	rs309577	T	A	0.65	-0.011	0.002	1.20E-08	0.65	-0.002	0.002	3.30E-01	0.87	0.0055
5	82878519	rs178024	G	A	0.65	-0.011	0.002	1.70E-08	0.66	-0.003	0.002	2.40E-01	0.85	0.0097
5	82882637	rs10646744	G	GAAAA	0.64	-0.011	0.002	3.60E-08	0.64	-0.002	0.002	3.20E-01		
5	82884891	rs310520	T	A	0.65	-0.011	0.002	1.10E-08	0.66	-0.003	0.002	2.50E-01	0.86	0.0082
5	82885467	5:82885467_ACTCT_A	ACTCT	A	0.65	-0.011	0.002	4.50E-08	0.65	-0.002	0.002	3.20E-01		
5	82886448	5:82886448_AAAC_A	AAAC	A	0.65	-0.011	0.002	4.60E-08	0.65	-0.002	0.002	3.20E-01		
5	82887835	rs4260646	A	G	0.65	-0.011	0.002	4.00E-08	0.65	-0.002	0.002	3.90E-01	0.87	0.0063
5	82887839	rs3864259	C	T	0.65	-0.011	0.002	4.00E-08	0.65	-0.002	0.002	3.80E-01	0.86	0.0066
5	82888812	rs310498	T	C	0.64	-0.011	0.002	3.20E-08	0.65	-0.003	0.002	2.80E-01	0.85	0.0097
5	82890586	rs753731735	ATCAG	A	0.66	-0.011	0.002	2.80E-08	0.66	-0.003	0.002	1.70E-01		
5	82891343	rs310502	A	T	0.65	-0.011	0.002	2.20E-08	0.65	-0.003	0.002	2.30E-01	0.84	0.0116
5	82891552	rs310503	G	T	0.66	-0.012	0.002	2.40E-09	0.66	-0.003	0.002	1.80E-01	0.86	0.0085
5	82896644	rs309563	A	T	0.66	-0.012	0.002	8.90E-10	0.66	-0.003	0.003	2.30E-01	0.88	0.0044
5	82898246	rs309570	T	G	0.66	-0.012	0.002	1.90E-09	0.67	-0.003	0.002	2.00E-01	0.86	0.0067
5	82899830	rs309574	C	T	0.66	-0.012	0.002	2.10E-09	0.67	-0.003	0.002	2.00E-01	0.86	0.0072
5	82900450	rs309575	C	A	0.66	-0.012	0.002	2.60E-09	0.67	-0.003	0.002	1.90E-01	0.86	0.0079
5	82902032	rs1673051	G	A	0.66	-0.012	0.002	1.40E-09	0.67	-0.003	0.002	2.90E-01	0.88	0.0035
5	82902267	rs12153581	A	T	0.66	-0.012	0.002	1.40E-09	0.67	-0.003	0.002	2.90E-01	0.88	0.0035
5	82904002	rs617228	C	G	0.66	-0.012	0.002	1.20E-09	0.67	-0.003	0.002	2.90E-01	0.88	0.0033
5	82905733	rs471580	C	T	0.66	-0.012	0.002	1.30E-09	0.67	-0.003	0.002	3.00E-01	0.88	0.0033
5	82909489	rs796444361	AC	A	0.64	-0.012	0.002	2.40E-09	0.65	-0.002	0.003	4.30E-01		

**5**	**82911748**	**rs492356**	**C**	**G**	**0.66**	**-0.012**	**0.002**	**2.00E-09**	0.66	-0.002	0.002	3.20E-01	0.88	**0.0033**
**5**	**82913284**	**rs567721**	**C**	**G**	**0.65**	**-0.011**	**0.002**	**4.40E-09**	0.65	-0.002	0.002	4.50E-01	0.89	**0.0023**
**5**	**82913361**	**rs566799**	**G**	**A**	**0.66**	**-0.012**	**0.002**	**1.80E-09**	0.66	-0.002	0.002	3.40E-01	0.89	**0.0028**
**5**	**82913780**	**rs651198**	**T**	**G**	**0.66**	**-0.012**	**0.002**	**1.70E-09**	0.66	-0.002	0.002	3.40E-01	0.89	**0.0028**
**5**	**82914527**	**rs336946**	**C**	**G**	**0.65**	**-0.012**	**0.002**	**3.90E-09**	0.65	-0.002	0.002	4.50E-01	0.89	**0.0022**
**5**	**82915184**	**rs336943**	**C**	**T**	**0.65**	**-0.012**	**0.002**	**3.90E-09**	0.65	-0.002	0.002	4.50E-01	0.89	**0.0022**
**5**	**82915497**	**rs336942**	**A**	**G**	**0.65**	**-0.012**	**0.002**	**3.90E-09**	0.65	-0.002	0.002	4.40E-01	0.89	**0.0023**
**5**	**82929987**	**rs9784716**	**C**	**A**	**0.67**	**-0.012**	**0.002**	**2.00E-09**	0.67	-0.003	0.002	2.70E-01	0.88	**0.0041**
**5**	**82932634**	**rs12189364**	**C**	**T**	**0.67**	**-0.011**	**0.002**	**1.10E-08**	0.68	-0.003	0.003	1.90E-01	0.84	**0.0117**
**5**	**82933835**	**rs71709944**	**C**	**CT**	**0.69**	**-0.013**	**0.002**	**1.20E-09**	0.69	-0.002	0.003	3.60E-01		
**5**	**82933913**	**rs17206069**	**G**	**A**	**0.67**	**-0.011**	**0.002**	**8.70E-09**	0.68	-0.003	0.003	1.90E-01	0.85	**0.0109**
**7**	**35428513**	**rs12155459**	**A**	**T**	0.77	-0.007	0.002	3.30E-03	**0.77**	**-0.015**	**0.003**	**4.20E-08**	0.84	**0.0135**
**7**	**35429465**	**rs11766731**	**G**	**T**	0.77	-0.007	0.002	2.80E-03	**0.77**	**-0.015**	**0.003**	**3.50E-08**	0.84	**0.0137**
**7**	**35438831**	**rs741408**	**T**	**C**	0.23	0.007	0.002	1.70E-03	**0.23**	**0.016**	**0.003**	**1.20E-08**	0.84	**0.0126**
**7**	**35440055**	**rs2592856**	**C**	**T**	0.23	0.007	0.002	1.80E-03	**0.23**	**0.016**	**0.003**	**6.90E-09**	0.85	**0.0099**
**7**	**35440155**	**rs11762074**	**T**	**C**	0.77	-0.007	0.002	1.50E-03	**0.77**	**-0.016**	**0.003**	**5.80E-09**	0.85	**0.0102**
**7**	**35446189**	**rs2592855**	**T**	**C**	0.23	0.007	0.002	1.30E-03	**0.23**	**0.016**	**0.003**	**9.70E-09**	0.84	**0.0131**
**7**	**35449334**	**rs998652**	**A**	**G**	0.23	0.007	0.002	1.40E-03	**0.23**	**0.016**	**0.003**	**1.00E-08**	0.84	**0.0130**
**7**	**35451830**	**rs17765314**	**G**	**A**	0.77	-0.007	0.002	1.40E-03	**0.77**	**-0.016**	**0.003**	**9.10E-09**	0.84	**0.0124**
**7**	**35451910**	**rs2592851**	**T**	**C**	0.23	0.007	0.002	1.40E-03	**0.23**	**0.016**	**0.003**	**9.00E-09**	0.84	**0.0124**
**7**	**35452092**	**rs1362655**	**G**	**A**	0.23	0.007	0.002	1.40E-03	**0.23**	**0.016**	**0.003**	**9.40E-09**	0.84	**0.0125**
**7**	**35455088**	**rs2592850**	**T**	**A**	0.32	0.007	0.002	4.50E-04	**0.32**	**0.014**	**0.003**	**4.60E-08**	0.77	**0.0376**
**7**	**35456691**	**rs2592849**	**A**	**T**	0.32	0.007	0.002	4.70E-04	**0.32**	**0.014**	**0.003**	**4.70E-08**	0.77	**0.0372**
**7**	**35457954**	**7:35457954_AT_A**	**AT**	**A**	0.32	0.007	0.002	8.40E-04	**0.32**	**0.014**	**0.003**	**1.60E-08**		
**7**	**35458137**	**rs140476889**	**A**	**AAAAC**	0.32	0.007	0.002	4.70E-04	**0.32**	**0.014**	**0.003**	**3.30E-08**		
**7**	**35459888**	**rs342977**	**G**	**A**	0.23	0.007	0.002	2.40E-03	**0.23**	**0.016**	**0.003**	**8.10E-09**	0.85	**0.0091**
**7**	**35460547**	**rs342979**	**C**	**T**	0.32	0.007	0.002	5.20E-04	**0.32**	**0.014**	**0.003**	**4.70E-08**	0.77	**0.0356**
**7**	**35461538**	**rs342981**	**G**	**A**	0.32	0.007	0.002	4.70E-04	**0.32**	**0.014**	**0.003**	**4.60E-08**	0.77	**0.0367**
**7**	**35461715**	**rs2551797**	**T**	**C**	0.32	0.007	0.002	4.80E-04	**0.32**	**0.014**	**0.002**	**4.70E-08**	0.77	**0.0368**
**7**	**35462145**	**rs342982**	**G**	**T**	0.32	0.007	0.002	4.80E-04	**0.32**	**0.014**	**0.003**	**4.50E-08**	0.77	**0.0363**

7	35466464	rs342986	G	A	0.23	0.007	0.002	2.30E-03	0.23	0.016	0.003	7.90E-09	0.85	0.0090
7	35466755	rs342987	T	G	0.23	0.007	0.002	2.30E-03	0.23	0.016	0.003	7.50E-09	0.85	0.0089
7	35467026	rs342988	C	T	0.28	0.007	0.002	7.40E-04	0.28	0.015	0.003	6.00E-09	0.83	0.0147
7	35467677	7:35467677_TAA_T	TAA	T	0.24	0.007	0.002	1.50E-03	0.24	0.016	0.003	1.80E-08		
7	35467896	rs342989	A	G	0.23	0.007	0.002	2.40E-03	0.23	0.016	0.003	7.00E-09	0.86	0.0085
7	35468763	rs17697812	T	C	0.77	-0.007	0.002	2.60E-03	0.77	-0.016	0.003	9.30E-09	0.85	0.0091
7	35469835	rs342992	C	T	0.23	0.007	0.002	2.60E-03	0.23	0.016	0.003	9.50E-09	0.85	0.0092
7	35477175	rs343000	C	T	0.23	0.007	0.002	2.40E-03	0.23	0.016	0.003	1.10E-08	0.85	0.0102
7	35478515	rs343002	T	C	0.23	0.007	0.002	2.40E-03	0.23	0.016	0.003	1.10E-08	0.85	0.0102
7	35479439	rs343003	C	T	0.23	0.007	0.002	2.40E-03	0.23	0.016	0.003	1.10E-08	0.85	0.0104
7	35488349	rs343011	T	G	0.23	0.007	0.002	2.30E-03	0.23	0.016	0.003	1.10E-08	0.85	0.0103
7	35496678	rs343026	T	G	0.32	0.007	0.002	7.70E-04	0.32	0.014	0.003	4.50E-08	0.79	0.0296
7	35497055	rs35099106	C	CT	0.23	0.007	0.002	2.50E-03	0.24	0.016	0.003	5.20E-09		
7	35498200	rs343029	A	G	0.23	0.007	0.002	2.20E-03	0.23	0.016	0.003	6.60E-09	0.85	0.0087
7	35500493	rs343031	G	A	0.32	0.007	0.002	7.70E-04	0.32	0.014	0.003	4.00E-08	0.79	0.0283
7	35503445	rs343036	G	C	0.23	0.007	0.002	2.20E-03	0.23	0.016	0.003	6.40E-09	0.86	0.0086
7	35503600	rs343037	G	T	0.23	0.007	0.002	2.20E-03	0.23	0.016	0.003	6.50E-09	0.86	0.0086
7	35503696	rs343038	G	C	0.32	0.007	0.002	8.10E-04	0.32	0.014	0.003	3.70E-08	0.80	0.0271
7	35505204	rs343039	C	T	0.23	0.007	0.002	2.60E-03	0.23	0.016	0.003	5.50E-09	0.86	0.0074
8	6470650	rs3020257	C	A	0.76	0.007	0.002	9.90E-04	0.76	0.016	0.003	2.50E-08	0.82	0.0187
8	6471126	rs2980664	G	A	0.76	0.008	0.002	4.80E-04	0.76	0.015	0.003	2.70E-08	0.80	0.0262
8	6472105	rs2959801	G	A	0.69	0.006	0.002	1.50E-03	0.69	0.015	0.003	2.00E-09	0.87	0.0063
8	6472603	rs11137038	G	A	0.64	0.007	0.002	2.80E-04	0.64	0.014	0.002	3.20E-08	0.76	0.0402
8	6473356	rs1256	C	G	0.70	0.007	0.002	3.40E-04	0.70	0.016	0.003	4.10E-10	0.86	0.0075
8	6473758	rs2454517	A	G	0.70	0.007	0.002	3.10E-04	0.70	0.016	0.003	1.40E-09	0.84	0.0121
8	6475489	rs2980662	G	C	0.68	0.007	0.002	8.80E-04	0.69	0.014	0.003	2.40E-08	0.81	0.0210
8	6476626	rs7005662	C	T	0.72	0.008	0.002	1.70E-04	0.73	0.015	0.003	8.60E-09	0.79	0.0286
8	6478481	rs2253560	G	A	0.72	0.008	0.002	1.80E-04	0.73	0.015	0.003	5.00E-09	0.80	0.0239
8	6478960	rs11137040	C	G	0.70	0.007	0.002	3.40E-04	0.71	0.014	0.003	3.30E-08	0.77	0.0355
8	6479432	rs2515559	A	G	0.72	0.008	0.002	2.60E-04	0.72	0.015	0.003	2.30E-08	0.77	0.0351

**8**	**6483059**	**rs201338894**	**ATAAAT**	**A**	0.72	0.008	0.002	1.10E-04	**0.73**	**0.016**	**0.003**	**2.40E-09**		
**8**	**6484505**	**8:6484505_CA_C**	**CA**	**C**	0.72	0.008	0.002	6.60E-05	**0.73**	**0.016**	**0.003**	**1.30E-09**		
**8**	**6484510**	**rs2454524**	**A**	**C**	0.72	0.008	0.002	6.70E-05	**0.73**	**0.016**	**0.003**	**1.30E-09**	0.81	**0.0219**
**8**	**6484530**	**rs2246801**	**C**	**T**	0.72	0.008	0.002	8.60E-05	**0.72**	**0.016**	**0.003**	**1.10E-09**	0.82	**0.0188**
**8**	**6484635**	**rs2278147**	**C**	**A**	0.71	0.008	0.002	1.90E-04	**0.71**	**0.017**	**0.003**	**3.00E-11**	0.88	**0.0037**
**8**	**6484982**	**rs2448611**	**C**	**T**	0.87	0.001	0.003	8.30E-01	**0.70**	**0.015**	**0.003**	**2.70E-08**		
**8**	**6485295**	**rs2912062**	**C**	**G**	0.70	0.008	0.002	1.60E-04	**0.71**	**0.017**	**0.003**	**3.10E-11**	0.88	**0.0041**
**8**	**6486033**	**rs2912063**	**A**	**G**	0.71	0.008	0.002	2.20E-04	**0.71**	**0.017**	**0.003**	**2.60E-11**	0.88	**0.0033**
**8**	**6487449**	**rs3020263**	**G**	**A**	0.72	0.008	0.002	1.00E-04	**0.72**	**0.016**	**0.003**	**7.40E-10**	0.83	**0.0152**
**8**	**6488577**	**rs3020264**	**G**	**A**	0.72	0.008	0.002	1.10E-04	**0.72**	**0.016**	**0.003**	**1.30E-09**	0.82	**0.0183**
**8**	**6488787**	**rs1974946**	**G**	**A**	0.72	0.008	0.002	1.10E-04	**0.72**	**0.016**	**0.003**	**1.40E-09**	0.82	**0.0191**
**8**	**6490200**	**rs2433143**	**C**	**T**	0.73	0.008	0.002	2.80E-04	**0.73**	**0.015**	**0.003**	**1.50E-08**	0.80	**0.0270**
**8**	**6490544**	**8:6490544_TA_T**	**TA**	**T**	0.70	0.007	0.002	7.00E-04	**0.71**	**0.016**	**0.003**	**1.80E-09**		
**19**	**45412079**	**rs7412**	**C**	**T**	0.92	0.016	0.003	4.40E-06	**0.92**	**0.027**	**0.004**	**4.40E-10**	0.75	**0.0458**
**19**	**45413233**	**rs1065853**	**G**	**T**	0.92	0.016	0.003	4.00E-06	**0.92**	**0.027**	**0.004**	**4.00E-10**	0.75	**0.0468**

Where: Insertion-deletion SNPs are not included due to software limitations.

Supplemental Table V: Genetic correlations with IMTmean by sex

	men					women				
	rg	se	z	p	FDR-P	rg	se	z	p	FDR-P
CHARGE IMTmean 2011	0.88	0.11	7.87	3.57E-15	**9.63E-14**	0.71	0.10	7.45	9.14E-14	**2.47E-12**
CHARGE IMTmean 2018	0.86	0.12	6.88	6.06E-12	**8.18E-11**	0.70	0.10	6.87	6.48E-12	**8.75E-11**
BMI	0.18	0.05	3.66	2.48E-04	**2.24E-03**	0.21	0.09	2.47	1.35E-02	9.14E-02
BMI men only	0.16	0.05	2.97	3.00E-03	**2.03E-02**					
BMI women only						0.13	0.05	2.54	0.01	9.99E-02
Fasting Insulin	0.31	0.11	2.71	6.70E-03	**3.02E-02**	0.23	0.10	2.36	1.84E-02	8.27E-02
Fasting Insulin men only	0.13	0.20	0.63	5.30E-01	7.95E-01					
Fasting Insulin women only						0.15	0.15	0.97	0.33	5.97E-01
Fasting Glucose	0.23	0.09	2.74	6.22E-03	**3.36E-02**	-0.12	0.05	-2.36	1.84E-02	9.91E-02
Fasting Glucose men only	0.00	0.15	-0.03	9.75E-01	9.75E-01					
Fasting Glucose women only						-0.06	0.13	-0.49	0.63	8.46E-01
Type 2 Diabetes	0.26	0.11	2.45	1.42E-02	5.50E-02	0.10	0.05	2.11	3.52E-02	1.36E-01
Rheumatoid arthritis	0.21	0.09	2.33	1.96E-02	6.60E-02	0.18	0.10	1.74	8.23E-02	2.78E-01
Ever Smoker	0.19	0.10	1.97	4.87E-02	1.46E-01	-0.10	0.07	-1.52	1.29E-01	3.48E-01
WHRadjBMI	0.12	0.07	1.75	8.08E-02	2.18E-01	0.03	0.02	1.36	1.74E-01	4.26E-01
WHRadjBMI men only	0.01	0.09	0.06	9.54E-01	1.03E+00					
WHRadjBMI women only						0.10	0.07	1.54	0.12	3.68E-01
Smoking onset	-0.36	0.22	-1.63	1.03E-01	2.53E-01	0.08	0.06	1.24	2.14E-01	4.82E-01
Fasting Proinsulin	0.28	0.18	1.57	1.18E-01	2.65E-01	0.03	0.02	1.06	2.88E-01	5.98E-01
HDL	-0.06	0.04	-1.28	2.00E-01	4.15E-01	-0.09	0.09	-1.04	3.00E-01	5.78E-01
Fasting Insulin adjBMI	0.10	0.11	0.90	3.69E-01	7.12E-01	-0.05	0.06	-0.86	3.89E-01	6.56E-01
Triglycerides	0.04	0.06	0.69	4.88E-01	7.76E-01	0.07	0.13	0.53	5.95E-01	8.46E-01
2hr Glucose	-0.10	0.14	-0.72	4.72E-01	7.97E-01	0.07	0.13	0.55	5.81E-01	8.71E-01
Ulcerative Collitis	-0.08	0.10	-0.75	4.56E-01	8.21E-01	0.03	0.05	0.59	5.56E-01	8.82E-01
Cholesterol	-0.02	0.07	-0.27	7.86E-01	9.23E-01	0.04	0.10	0.40	6.87E-01	7.42E-01
LDL	0.02	0.06	0.30	7.66E-01	9.41E-01	-0.12	0.27	-0.43	6.67E-01	7.50E-01

Ciggarettes per day	0.05	0.14	0.33	7.38E-01	9.49E-01	-0.03	0.07	-0.45	6.52E-01	7.65E-01
DBP	0.01	0.03	0.38	7.06E-01	9.53E-01	0.07	0.16	0.46	6.46E-01	7.92E-01
Irritatable Bowel Disorder	-0.03	0.08	-0.41	6.85E-01	9.74E-01	-0.04	0.09	-0.49	6.28E-01	8.07E-01
Cardiovascular disease	-0.05	0.30	-0.16	8.72E-01	9.81E-01	-0.02	0.08	-0.26	7.96E-01	8.27E-01
SBP	0.00	0.03	-0.05	9.56E-01	9.93E-01	-0.01	0.17	-0.04	9.69E-01	9.69E-01

Supplemental Table VI: Previous associations with SNPs in GWAS-significant loci

Chr	BP	SNP	RA	RAF	OR	Beta	P-value	Mapped gene	P-value annotation	A1	IMTmean Beta	IMTmean P	direction	PMID
5	81696676	rs446064					9 x 10-29	*SSBP2*	Adolescent idiopathic scoliosis					30019117
7	35428286	rs342989	A	0.23		0.1631 mmHg increase	3 x 10-15	*AC007652.1, AC009531.1*	Diastolic blood pressure	A	0.01	3.6E-11	consistent	30224653
7	35435712	rs2541918	T	0.68		0.1567 unit decrease	8 x 10-7	*AC007652.1, AC009531.1*	Diastolic blood pressure	A	0.01	8.7E-08	consistent	28135244
7	35506177	rs11763856	T	0.03		3.1 ms increase	4 x 10-10	*AC007652.1*	PR interval	C	-0.02	0.00022		30046033
7	35506177	rs11763856	T	0.04		3.055 unit increase	1 x 10-8	*AC007652.1*	PR interval	C	-0.02	0.00022		30679814
7	35515178	rs343064	A	0.40		1.31 unit increase	3 x 10-8	*HERPUD2, AC007652.1*	Non-alcoholic fatty liver disease histology (Fibrosis)	C	0.00	0.96		20708005
8	6493731	rs17077154	G	0.07		0.302 unit increase	8 x 10-6	*MCPH1*	Cytomegalovirus antibody response					21993531
8	6494184	rs141416541	T	0.03		0.4511 unit decrease	4 x 10-7	*MCPH1*	Diastolic blood pressure	C	0.01	0.0019	consistent	28135244
10	30167199	rs2779071					2 x 10-11	*RNU6-598P, JCAD*		T	0.00	5.40E-01		30595370
11	103718366	rs1917445					6 x 10-7	*AP002989.1*	Neutrophil count	C	0.01	2.6E-06		21507922
11	103789839	rs974819	T	0.32	1.07		2 x 10-9	*AP002989.1*	Coronary heart disease	T	0.01	5.6E-10	consistent	21378988
11	103789839	rs974819	T	0.31		0.0614 unit increase	1 x 10-28	*AP002989.1*	Coronary artery disease	T	0.01	5.6E-10	consistent	29212778

11	103789839	rs974819	T	0.31		0.0674 unit increase	6 x 10-29	*AP002989.1*	Coronary artery disease	T	0.01	5.6E-10	consistent	29212778
11	103798234	rs2019090	A	0.36	1.07		4 x 10-9	*AP002989.1*	Myocardial infarction	A	0.01	3.7E-10	consistent	26343387
13	110397276	rs9515203	T	0.74		0.068 unit increase	6 x 10-24	*COL4A2*	Coronary artery disease	T	-0.01	1.1E-08	consistent	29212778
13	110397276	rs9515203	T	0.74		0.0596 unit increase	4 x 10-23	*COL4A2*	Coronary artery disease	T	-0.01	1.1E-08	consistent	29212778
13	110397276	rs9515203	T	0.74		0.0675 unit increase	6 x 10-24	*COL4A2*	Coronary artery disease	T	-0.01	1.1E-08	consistent	29212778
19	41319286	rs73045269	T	0.15		0.07 unit increase	1 x 10-14	*CCDC97, TGFB1*	Coronary artery disease	C	0.00	0.99		29212778
19	41326326	rs12980942	A	0.16		0.0609 unit decrease	7 x 10-15	*TGFB1*	Coronary artery disease	G	0.00	0.29		29212778
19	41345604	rs4803455	A	0.49		0.054 unit decrease	4 x 10-17	*TGFB1*	Coronary artery disease	C	0.00	0.19		29212778
19	41345604	rs4803455	A	0.50		0.0484 unit decrease	2 x 10-17	*TGFB1*	Coronary artery disease	C	0.00	0.19		29212778
19	41345604	rs4803455	A	0.50		0.1617 mmHg decrease	8 x 10-11	*TGFB1*	Pulse pressure	C	0.00	0.19		30578418
19	41346870	rs2288874	T	0.42		0.0531 unit increase	4 x 10-16	*TGFB1*	Coronary artery disease	T	0.00	0.11		29212778
19	41348629	rs8108632	T	0.48	1.05		4 x 10-8	*TGFB1*	Coronary artery disease (broad definition)	T	0.00	0.2		28714975
19	41353016	rs1800470	A	0.62		0.259 unit increase	5 x 10-26	*TGFB1, AC011462.1, TMEM91*	Blood protein levels (TGFB1)	G	0.00	0.33		30072576

19	41355454	rs4803457	T		0.149 unit increase	2 x 10-8	*B9D2, TMEM91, AC011462.1*	Pulse pressure	T	0.00	0.32		30595370
19	44908822	rs7412			0.75 unit decrease	3 x 10-58	*APOE*	Lipid metabolism phenotypes (L-LDL-FC)	C	0.02	2.1E-15		22286219
19	44908822	rs7412			6.2 mg/dL decrease	2 x 10-47	*APOE*	Response to statins (baseline LDL-C)	C	0.02	2.1E-15		22331829
19	44908822	rs7412		0.10	12.3 mg/dL decrease	2 x 10-9	*APOE*	LDL cholesterol	C	0.02	2.1E-15		23067351
19	44908822	rs7412	T	0.12	0.48 unit decrease	3 x 10-53	*APOE*	Lipid traits (LDL-C)	C	0.02	2.1E-15	consistent	24023260
19	44908822	rs7412	T	0.12	0.09 unit decrease	2 x 10-30	*APOE*	Lipid traits (TC)	C	0.02	2.1E-15	consistent	24023260
19	44908822	rs7412	C	0.93	0.413 s.d. increase	8 x 10-239	*APOE*	Cholesterol, total	C	0.02	2.1E-15	consistent	25961943
19	44908822	rs7412				9 x 10-16	*APOE*	Ideal cardiovascular health (clinical and behavioural)	C	0.02	2.1E-15		27179730
19	44908822	rs7412	T	0.06	0.59 unit decrease	2 x 10-120	*APOE*	Metabolite levels (Free cholesterol in large LDL)	C	0.02	2.1E-15	consistent	27005778
19	44908822	rs7412	T	0.08	0.1564597 mg/dl increase	6 x 10-14	*APOE*	HDL cholesterol	C	0.02	2.1E-15	consistent	28270201
19	44908822	rs7412	T	0.08	0.41957754 mg/dl decrease	5 x 10-94	*APOE*	Cholesterol, total	C	0.02	2.1E-15	consistent	28270201

19	44908822	rs7412	T	0.07		0.59 unit decrease	2 x 10-286	*APOE*	LDL cholesterol levels (Trans-ethnic initial)	C	0.02	2.1E-15	consistent	28334899
19	44908822	rs7412	T	0.07		0.374 unit decrease	8 x 10-315	*APOE*	Total cholesterol levels (Trans-ethnic initial)	C	0.02	2.1E-15	consistent	28334899
19	44908822	rs7412	T			0.313 mmol/L decrease	7 x 10-15	*APOE*	LDL cholesterol levels	C	0.02	2.1E-15	consistent	28371326
19	44908822	rs7412	T	0.08		0.3826 unit decrease	4 x 10-10	*APOE*	Pulse pressure	C	0.02	2.1E-15	consistent	28135244
19	44908822	rs7412	T			3.34 mg/dL decrease	3 x 10-10	*APOE*	Lipoprotein (a) levels	C	0.02	2.1E-15		28512139
19	44908822	rs7412	T			2.78 unit decrease	3 x 10-9	*APOE*	Lipoprotein(a) levels adjusted for apolipoprotein(a) isoforms	C	0.02	2.1E-15	consistent	28512139
19	44908822	rs7412	T	0.08		0.419 unit decrease	3 x 10-19	*APOE*	Low density lipoprotein cholesterol levels	C	0.02	2.1E-15	consistent	28548082
19	44908822	rs7412	T	0.08		0.27 unit decrease	1 x 10-8	*APOE*	Total cholesterol levels	C	0.02	2.1E-15	consistent	28548082
19	44908822	rs7412				11.96 unit increase	9 x 10-19	*APOE*	Cardiovascular risk factors (LDL-cholesterol)	C	0.02	2.1E-15		20838585
19	44908822	rs7412	C	0.92	1.15		2 x 10-19	*APOE*	Coronary artery disease (broad definition)	C	0.02	2.1E-15	consistent	28714975

19	44908822	rs7412	T	0.06		17.11 (nmol/min/ml) decrease	8 x 10-78	*APOE*	Lipoprotein phospholipase A2 activity in cardiovascular disease	C	0.02	2.1E-15	consistent	28753643
19	44908822	rs7412	T	0.06		0.04 unit decrease	2 x 10-27	*APOE*	Lipoprotein-associated phospholipase A2 activity change in response to darapladib treatment in cardiovascular disease	C	0.02	2.1E-15	consistent	28753643
19	44908822	rs7412	T	0.08		0.16 unit decrease	3 x 10-39	*APOE*	Coronary artery disease	C	0.02	2.1E-15	consistent	29212778
19	44908822	rs7412	T	0.08		0.1368 unit decrease	2 x 10-35	*APOE*	Coronary artery disease	C	0.02	2.1E-15	consistent	29212778
19	44908822	rs7412	T	0.08		0.1603 unit decrease	3 x 10-39	*APOE*	Coronary artery disease	C	0.02	2.1E-15	consistent	29212778
19	44908822	rs7412			2.31		3 x 10-105	*APOE*	Alzheimer's disease (late onset)	C	0.02	2.1E-15		28714976
19	44908822	rs7412					9 x 10-62	*APOE*	Family history of Alzheimer's disease	C	0.02	2.1E-15		29777097
19	44908822	rs7412					2 x 10-76	*APOE*	Alzheimer's disease or family history of Alzheimer's disease	C	0.02	2.1E-15		29777097

19	44908822	rs7412	C	0.94	0.795 unit increase	3 x 10-56	*APOE*	Blood protein levels (APOE)	C	0.02	2.1E-15	consistent	30072576
19	44908822	rs7412		0.08	0.3753 unit decrease	5 x 10-31	*APOE*	Pulse pressure	C	0.02	2.1E-15		30224653
19	44908822	rs7412				3 x 10-63	*APOE*	Cardiovascular disease	C	0.02	2.1E-15		30595370
19	44908822	rs7412				4 x 10-21	*APOE*	Systolic blood pressure	C	0.02	2.1E-15		30595370
19	44908822	rs7412	C	0.92	0.079 unit decrease	5 x 10-29	*APOE*	High density lipoprotein cholesterol levels	C	0.02	2.1E-15	consistent	29507422
19	44908822	rs7412	C	0.95	0.152 unit decrease	2 x 10-7	*APOE*	High density lipoprotein cholesterol levels	C	0.02	2.1E-15	consistent	29507422
19	44908822	rs7412	C		0.085 unit decrease	2 x 10-36	*APOE*	High density lipoprotein cholesterol levels	C	0.02	2.1E-15	consistent	29507422
19	44908822	rs7412	C	0.92	0.493 unit increase	1 x 10-300	*APOE*	Low density lipoprotein cholesterol levels (EA)	C	0.02	2.1E-15	consistent	29507422
19	44908822	rs7412	C	0.95	0.574 unit increase	9 x 10-59	*APOE*	Low density lipoprotein cholesterol levels (Hispanic)	C	0.02	2.1E-15	consistent	29507422
19	44908822	rs7412	C	0.89	0.522 unit increase	2 x 10-36	*APOE*	Low density lipoprotein cholesterol levels (AA)	C	0.02	2.1E-15	consistent	29507422

19	44908822	rs7412	C			0.498 unit increase	1 x 10-300	*APOE*	Low density lipoprotein cholesterol levels	C	0.02	2.1E-15	consistent	29507422
19	44908822	rs7412	C	0.92		0.09 unit decrease	5 x 10-31	*APOE*	Triglycerides (EA)	C	0.02	2.1E-15	**inconsitent**	29507422
19	44908822	rs7412	C			0.087 unit decrease	2 x 10-32	*APOE*	Triglycerides	C	0.02	2.1E-15	**inconsitent**	29507422
19	44908822	rs7412	C	0.92		0.342 unit increase	1 x 10-300	*APOE*	Total cholesterol levels (EA)	C	0.02	2.1E-15	consistent	29507422
19	44908822	rs7412	C	0.95		0.411 unit increase	3 x 10-31	*APOE*	Total cholesterol levels (Hispanic)	C	0.02	2.1E-15	consistent	29507422
19	44908822	rs7412	C	0.89		0.381 unit increase	3 x 10-20	*APOE*	Total cholesterol levels (AA)	C	0.02	2.1E-15	consistent	29507422
19	44908822	rs7412	C			0.348 unit increase	1 x 10-300	*APOE*	Total cholesterol levels	C	0.02	2.1E-15	consistent	29507422
19	44908822	rs7412	T	0.08		0.3255 mmHg decrease	2 x 10-11	*APOE*	Systolic blood pressure	C	0.02	2.1E-15	consistent	30578418
19	44908822	rs7412	T	0.08		0.3606 mmHg decrease	4 x 10-24	*APOE*	Pulse pressure	C	0.02	2.1E-15	consistent	30578418
19	44908822	rs7412	T	0.08		21.199877 z-unit decrease	1 x 10-99	*APOE*	Alzheimer's disease or family history of Alzheimer's disease	C	0.02	2.1E-15		30617256
19	44908822	rs7412	T	0.08		0.0119 unit decrease	1 x 10-14	*APOE*	Carotid intima media thickness	C	0.02	2.1E-15	consistent	30510157
19	44908822	rs7412	C	0.93	2.07		1 x 10-7	*APOE*	Alzheimer's disease (females)	C	0.02	2.1E-15		30636644
19	44908822	rs7412	C	0.93	2.21		4 x 10-13	*APOE*	Alzheimer's disease	C	0.02	2.1E-15		30636644

19	44909976	rs1065853	G	0.93	0.603 s.d. increase	5 x 10-324	*AC011481.3*	LDL cholesterol	G	0.02	1.6E-15	consistent	25961943
19	44909976	rs1065853			0.573 unit decrease	5 x 10-50	*AC011481.3*	Total cholesterol levels in LDL	G	0.02	1.6E-15		29084231
19	44909976	rs1065853			0.399 unit decrease	4 x 10-25	*AC011481.3*	Serum total cholesterol levels	G	0.02	1.6E-15		29084231
19	44909976	rs1065853			0.642 unit decrease	1 x 10-62	*AC011481.3*	Free cholesterol levels in large LDL	G	0.02	1.6E-15		29084231
19	44909976	rs1065853	T	0.08	0.72 unit decrease	2 x 10-58	*AC011481.3*	Blood protein levels ((isoform E3), APOE)	G	0.02	1.6E-15	consistent	29875488
19	44909976	rs1065853	T	0.06	13.1251 unit decrease	2 x 10-26	*AC011481.3*	Total cholesterol levels	G	0.02	1.6E-15	consistent	30718733
19	44909976	rs1065853	T		0.1615 unit decrease	3 x 10-54	*AC011481.3*	Low density lipoprotein cholesterol levels	G	0.02	1.6E-15	consistent	30718733

Supplemental Table VII: Predicted functional, coding or loss of function variants

#Uploaded_variation	chr	pos	Allele	Consequence	IMPACT	SYMBOL	SIFT	PolyPhen
rs6658335	1	1707555	T	missense_variant	MODERATE	*CDK11A*	tolerated_low_confidence(0.06)	benign(0.007)
rs2912010	8	6621465	T	synonymous_variant	LOW	*MCPH1*	-	-
rs1057090	8	6621521	A	missense_variant	MODERATE	*MCPH1*	tolerated(0.08)	benign(0.129)
rs1057090	8	6621521	G	missense_variant	MODERATE	*MCPH1*	deleterious(0.02)	benign(0.025)
rs1057090	8	6621521	T	missense_variant	MODERATE	*MCPH1*	tolerated(1)	benign(0)
rs2912016	8	6621657	A	synonymous_variant	LOW	*MCPH1*	-	-
rs2912057	8	6625961	G	missense_variant	MODERATE	*MCPH1*	-	-
rs2912057	8	6625961	G	missense_variant	MODERATE	*MCPH1*	-	-
rs11558719	10	72213199	T	missense_variant	MODERATE	*ASCC1*	tolerated(0.21)	benign(0.203)
rs28375406	16	88930433	G	missense_variant	MODERATE	*LOC105371409*	-	-
rs12924185	16	88950476	T	missense_variant	MODERATE	*LOC100129697*	tolerated_low_confidence(0.07)	unknown(0)
rs1160983	19	44893972	A	synonymous_variant	LOW	*TOMM40*	-	-
rs7412	19	44908822	T	missense_variant	MODERATE	*APOE*	deleterious(0)	probably_damaging(1)

Supplemental Table VIII: EQTLS identified in GTEx

	SNP Id	Chr	Pos	A1	Gene Symbol	Gencode Id	P-Value	NES	Tissue
lead	rs309563	5	82896644	A	*VCAN-AS1*	ENSG00000249835.2	4.60E-06	-0.2	Testis
VEP	rs1057090	8	6479042	C	*MCPH1-AS1*	ENSG00000271743.1	5.20E-06	-0.44	Testis
VEP	rs1057090	8	6479042	C	*MCPH1-AS1*	ENSG00000271743.1	2.70E-05	-0.26	Thyroid
VEP	rs2912010	8	6478986	C	*MCPH1-AS1*	ENSG00000271743.1	8.30E-06	-0.28	Nerve - Tibial
VEP	rs2912010	8	6478986	C	*MCPH1-AS1*	ENSG00000271743.1	8.00E-06	-0.43	Testis
VEP	rs2912016	8	6479178	C	*MCPH1-AS1*	ENSG00000271743.1	3.90E-06	-0.37	Colon - Transverse
VEP	rs2912016	8	6479178	C	*MCPH1-AS1*	ENSG00000271743.1	1.30E-05	-0.3	Esophagus - Muscularis
VEP	rs2912016	8	6479178	C	*MCPH1-AS1*	ENSG00000271743.1	4.30E-09	-0.37	Nerve - Tibial
VEP	rs2912016	8	6479178	C	*MCPH1-AS1*	ENSG00000271743.1	3.10E-06	-0.58	Prostate
VEP	rs2912016	8	6479178	C	*MCPH1-AS1*	ENSG00000271743.1	3.90E-06	-0.46	Stomach
VEP	rs2912016	8	6479178	C	*MCPH1-AS1*	ENSG00000271743.1	2.80E-06	-0.47	Testis
VEP	rs2912016	8	6479178	C	*MCPH1-AS1*	ENSG00000271743.1	1.00E-07	-0.33	Thyroid
VEP	rs2912057	8	6483482	C	*MCPH1-AS1*	ENSG00000271743.1	2.30E-05	-0.26	Nerve - Tibial
lead	rs2912062	8	6485295	C	*MCPH1-AS1*	ENSG00000271743.1	3.10E-06	-0.52	Brain - Cortex
lead	rs2912062	8	6485295	C	*MCPH1-AS1*	ENSG00000271743.1	7.70E-07	-0.46	Brain - Nucleus accumbens (basal ganglia)
lead	rs2912062	8	6485295	C	*MCPH1-AS1*	ENSG00000271743.1	1.20E-06	-0.65	Cells - EBV-transformed lymphocytes
lead	rs2912062	8	6485295	C	*MCPH1-AS1*	ENSG00000271743.1	5.40E-11	-0.55	Colon - Transverse
lead	rs2912062	8	6485295	C	*MCPH1-AS1*	ENSG00000271743.1	1.60E-06	-0.35	Esophagus - Muscularis
lead	rs2912062	8	6485295	C	*MCPH1-AS1*	ENSG00000271743.1	8.70E-11	-0.43	Nerve - Tibial
lead	rs2912062	8	6485295	C	*MCPH1-AS1*	ENSG00000271743.1	2.70E-06	-0.58	Ovary
lead	rs2912062	8	6485295	C	*MCPH1-AS1*	ENSG00000271743.1	2.10E-06	-0.48	Stomach
lead	rs2912062	8	6485295	C	*MCPH1-AS1*	ENSG00000271743.1	4.80E-08	-0.55	Testis
lead	rs2912062	8	6485295	C	*MCPH1-AS1*	ENSG00000271743.1	2.40E-07	-0.34	Thyroid
lead	rs2912063	8	6486033	A	*MCPH1-AS1*	ENSG00000271743.1	4.60E-06	-0.51	Brain - Cortex
lead	rs2912063	8	6486033	A	*MCPH1-AS1*	ENSG00000271743.1	2.00E-06	-0.44	Brain - Nucleus accumbens (basal ganglia)
lead	rs2912063	8	6486033	A	*MCPH1-AS1*	ENSG00000271743.1	8.40E-11	-0.55	Colon - Transverse
lead	rs2912063	8	6486033	A	*MCPH1-AS1*	ENSG00000271743.1	3.80E-07	-0.37	Esophagus - Muscularis

lead	rs2912063	8	6486033	A	*MCPH1-AS1*	ENSG00000271743.1	6.20E-10	-0.41	Nerve - Tibial
lead	rs2912063	8	6486033	A	*MCPH1-AS1*	ENSG00000271743.1	2.90E-06	-0.59	Ovary
lead	rs2912063	8	6486033	A	*MCPH1-AS1*	ENSG00000271743.1	1.20E-06	-0.5	Stomach
lead	rs2912063	8	6486033	A	*MCPH1-AS1*	ENSG00000271743.1	1.10E-07	-0.54	Testis
lead	rs2912063	8	6486033	A	*MCPH1-AS1*	ENSG00000271743.1	3.30E-07	-0.34	Thyroid
Lead	rs2150562	10	29873494	A	*SVIL*	ENSG00000197321.14	3.30E-13	-0.16	Artery - Tibial
Lead	rs2150562	10	29873494	A	*SVIL*	ENSG00000197321.14	9.4E-06	-0.13	Artery - Aorta
Lead	rs2150562	10	29873494	A	*SVIL*	ENSG00000197321.14	0.00011	-0.13	Thyroid
lead	rs2019090	11	103668962	A	*RP11-563P16.1*	ENSG00000254987.1	3.70E-05	-0.3	Artery - Aorta
lead	rs561732	16	88988989	T	*CBFA2T3*	ENSG00000129993.10	7.10E-15	-0.47	Pancreas
lead	rs561732	16	88988989	T	*CBFA2T3*	ENSG00000129993.10	1.90E-05	-0.17	Thyroid
VEP	rs28375406	16	88996841	A	*CBFA2T3*	ENSG00000129993.10	2.90E-10	-0.39	Pancreas
VEP	rs28375406	16	88996841	A	*CBFA2T3*	ENSG00000129993.10	1.50E-05	-0.17	Thyroid
VEP	rs7412	19	45412079	C	*APOE*	ENSG00000130203.5	2.20E-05	-0.29	Skin - Not Sun Exposed (Suprapubic)

Where: *CTD-2541M15.3* also known as *MCPH1-AS1*

Supplemental Table IX: Baseline demographic characteristics of the individuals included in the analysis of ISH

	ISH	Controls
N (%)	7944 (71.8)	367952 (45.1)
Age (years)	62.2 (5.9)	56.8 (8.0)
Weight (Kg)	82.5 (15.2)	78.2 (15.9)
Waist (cm)	96.0 (12.5)	90.3 (13.5)
Hip (cm)	104.0 (8.6)	103.5 (9.2)
WHR	0.92 (0.08)	0.87 (0.09)
BMI (kg/m2)	28.4 (4.5)	27.4 (4.8)
SBP (mmHg)	134 (17)	139 (19)
DBP (mmHg)	77 (10)	82 (10)
Corrected SBP (mmHg)*	141 (18)	142 (21)
Corrected DBP (mmHg)*	82 (10)	84 (11)
N hypertension	2680 (36.2)	190044 (54.2)
N on BPmedication	3398 (43.4)	74176 (20.3)
N on lipid lowering medication	4854 (89.1)	33456 (22.8)
N probable t2d	784 (9.9)	15422 (4.2)
N curent smoking	1033 (13.1)	37428 (10.2)

Where: * adjusted for blood pressure medication as per Ehret et al, T2D, type 2 diabetes; ISH, ischemic heart disease

Supplemental Table X: Baseline demographic characteristics of the individuals included in the analysis of stroke

	Stroke	Controls
N (% men)	6060 (59.8)	367945 (45.1)
Age (years)	61.3 (6.5)	56.8 (8.0)
Weight (Kg)	82.5 (16.6)	78.2 (15.9)
Waist (cm)	96.5 (13.9)	90.3 (13.5)
Hip (cm)	105.3 (9.9)	103.5 (9.2)
WHR	0.92 90.09)	0.87 (0.09)
BMI (kg/m2)	29.0 (5.1)	27.4 (4.8)
SBP (mmHg)	140 (19)	139 (19)
DBP (mmHg)	82 (10)	82 (10)
Corrected SBP (mmHg)*	149 (21)	142 (21)
Corrected DBP (mmHg)*	88 (12)	84 (11)
N hypertension	4259 (72.6)	190038 (54.2)
N on BPmedication	3726 (62.2)	74203 (20.3)
N on lipid lowering medication	2739 (86.4)	33470 (22.8)
N probable t2d	810 (13.4)	15446 (4.2)
n curent smoking	963 (16.0)	37445 (10.2)

Where: * adjusted for blood pressure medication as per Ehret et al, T2D, type 2 diabetes; ISH, ischemic heart disease

Supplemental Table XI: Effect of IMT SNPs on ISH or stroke in UKB

		cIMT			Stroke						ISH				
Analysis	SNP	CHR	A1	beta	A1	N	OR	L95	U95	P	N	OR	L95	U95	P
IMTmean sex combined	rs758080886	5	CA	0.013	CA	299237	0.92	0.87	0.99	0.0170	300879	0.93	0.88	0.98	0.0091
	rs342988	7	C	0.011	C	368953	1.04	1.00	1.08	0.0776	370860	1.01	0.97	1.04	0.7248
	rs2912062	8	C	0.012	G	361917	0.96	0.92	1.00	0.0618	363800	0.97	0.94	1.01	0.1172
	rs34557926	8	C	-0.011	T	364824	1.00	0.96	1.04	0.8961	366699	1.00	0.96	1.03	0.7896
	rs2019090	11	A	0.009	A	369140	1.02	0.97	1.06	0.4789	371023	1.09	1.05	1.13	**2.73E-06**
	rs561732	16	T	0.009	C	363617	1.01	0.97	1.05	0.7940	365499	0.97	0.93	1.00	0.0560
	rs111689747	19	G	-0.044	A	368879	0.96	0.80	1.16	0.6831	370787	1.00	0.85	1.17	0.9954
	rs1065853	19	G	0.021	T	370443	0.91	0.85	0.97	0.0064	372354	0.83	0.78	0.88	**3.07E-09**
IMTmax sex combined	rs11762074	7	T	-0.014	C	369523	1.01	0.96	1.05	0.8068	371440	1.01	0.97	1.04	0.7928
	rs11025608	11	G	-0.010	G	362725	1.01	0.98	1.05	0.4502	364596	1.01	0.98	1.04	0.6616
IMTmeanmax sex combined	rs2150562	10	A	0.008	A	370859	1.03	0.99	1.066	0.2207	372774	1.00	0.96	1.033	0.9018
IMTmean Men	rs35099106	7	C	0.016	C	367661	1.01	0.96	1.05	0.8134	369559	1.00	0.96	1.04	0.9698
	rs2912063	8	A	0.017	G	361633	0.96	0.92	1.00	0.0595	363507	0.98	0.94	1.01	0.1665
IMTmean Women	rs309563	5	A	-0.012	T	363632	1.00	0.96	1.04	0.9140	365516	1.02	0.98	1.05	0.3707

Supplemental Table XII: Quality control data for lead SNPs.

Analysis	SNP	CHR	locus*	A1	A0	A1 freq	GWAS SNPs in locus	Missing	N A1 het	N homo	N A0 het	Observed het	Expected het	HWE P
IMTmean sex combined	rs758080886	5		CA	C	0.15	1	0.189	231	3851	13879	0.214	0.211	0.0520
	rs342988†||	7	35423859-35528567	C	T	0.28	78	0.004	1719	8855	11470	0.402	0.402	0.8670
	rs2912062‡	8	6470650-6494850	C	G	0.71	56	0.023	10803	8993	1843	0.416	0.414	0.6459
	rs34557926||	8	124579985-124608614	C	T	0.64	4	0.016	8969	10028	2788	0.460	0.460	0.8596
	rs2019090||	11	103660567-103673294	A	T	0.29	5	0.003	1852	9036	11180	0.410	0.411	0.6700
	rs561732	16	88966667-88989862	T	C	0.65	22	0.018	9216	9797	2728	0.451	0.456	0.1179
	rs111689747||	19	41188985-41371458	G	A	0.99	3	0.006	21527	489	3	0.022	0.022	0.7587
	rs1065853§	19	45412079-45426792	G	T	0.92	7	0.001	18790	3201	130	0.145	0.144	0.6413
IMTmax sex combined	rs11762074 †	7	35428513-35505204	T	C	0.77	63	0.004	12974	7883	1207	0.357	0.358	0.8360
	rs11025608||	11		G	T	0.44	1	0.022	4156	10839	6659	0.501	0.493	0.0316
	rs1065853§	19	45389596-45426792	G	T	0.92	11	as above						
IMTmeanmax sex combined	rs2150562||	10	30161168-30168699	A	G	0.311	4	0	2155	9481	10507	0.428	0.429	0.8142
IMTmean Men	rs35099106 †	7	35428513-35505204	C	CT	0.24	38	0.008	1203	7843	12914	0.357	0.358	0.7917

	rs2912063‡	8	6470650-6490544	A	G	0.71	24	0.023	10919	8915	1799	0.412	0.411	0.7408
	rs1065853§	19	45412079-45413233	G	T	0.92	2	as above						
IMTmean Women	rs309563||	5	82870033-82933913	A	T	0.659	37	0.019	9549	9723	2453	0.448	0.447	0.7729

Where: *, region bordered by gwas-significant SNPs; het, heterozygotes; homo, homozygotes; HWE P, Pvalue for test of deviation from Hardy-Weinberg equilibrium; †, LD between rs342988, rs11762074 and rs35099106 suggests that these SNPs repesent 1 signal; ‡, LD between rs2912062 and rs2912063 indicate that these SNPs are proxies; §, consistent lead SNP convincingly demonstrates that the same locus is being identified; ||, novel locus.
