# Supplementary figures and images for: Carotid Intima-Media Thickness: Novel Loci, Sex-Specific Effects, and Genetic Correlations With Obesity and Glucometabolic Traits in UK Biobank
Source: Arterioscler Thromb Vasc Biol. 2019 Dec 5;40(2):446–61. doi: 10.1161/ATVBAHA.119.313226 (PMC6975521; doi:10.1161/ATVBAHA.119.313226)

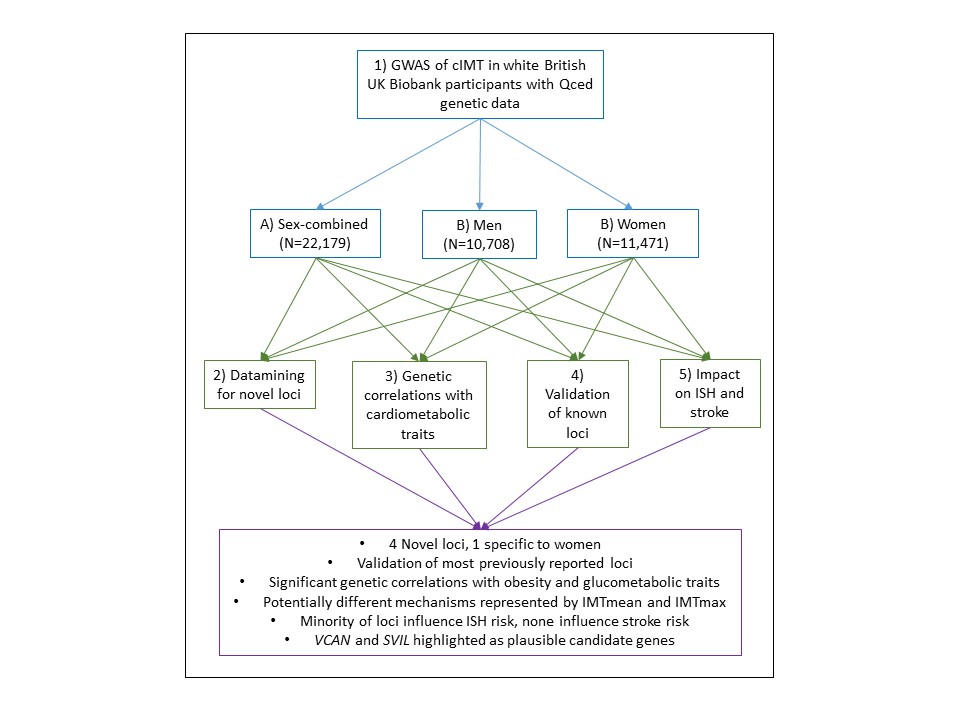

Supplement: Supplementary file 2 [file atv-40-446-s002.jpg]
